# Thermodynamics of Interfacial Water Confinement Reveal Altered Mechanical Properties in Polysaccharides Films

**DOI:** 10.1021/acspolymersau.6c00003

**Published:** 2026-03-16

**Authors:** Hojin Seo, Xiaoqing Yu, Tequila A. L. Harris

**Affiliations:** George W. Woodruff School of Mechanical Engineering, 1372 Georgia Institute of Technology, Atlanta, Georgia 30332-0405, United States

**Keywords:** Polymer mechanics, interfacial water, disjoining pressure, steric effects, chitosan

## Abstract

In this work, we introduce a water-driven thermodynamic mechanism, herein referred to as hydro-softening, rendering polysaccharide films soft and stretchable during thin-film processing. Distinct from conventional water-mediated plasticization or swelling, we show that substrate effects during processing can reorganize hydration clusters on the polymer chain, generating disjoining pressure and penalizing configurational entropy, with mechanical softening thereby arising as a consequence. Chemical, thermal, and mechanical characterization corroborates the presence of confined water molecules as the primary driver of hydro-softening. Notably, we observed distinct cold crystallization of confined water under hydro-softened conditions. We also found greater mass loss at temperatures above 100 °C—the boiling point of unbound water—coupled with pronounced decreases in both elastic modulus and hardness under hydro-softened conditions. Furthermore, a physically informed simulation is introduced to structurally model the emergent confined water, qualitatively revealing how steric confinement alters the hydration landscape, leading to anisotropic hydration clustering. Although water-induced softening has long been observed in polymers, the absence of mechanistic insight has constrained the development of functional materials. The thermodynamic framework established here addresses this limitation, enabling a more deliberate and extensible application of these systems.

## Graphical abstract

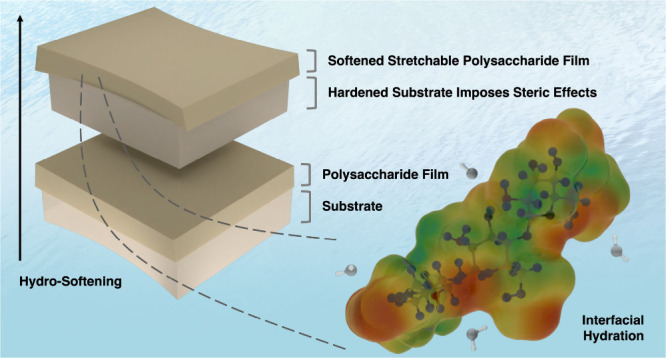

## Introduction

Mechanical properties of polysaccharides are sensitive to their hydration state; however, the thermodynamic principles governing this dependence on interfacial water structure remain incompletely understood.^1,2^ In this work, distinctions between unbound, weakly bound, and tightly bound water are defined operationally based on their experimental signatures, consistent with prior literature.^3,4^ In particular, tightly bound water is characterized by the absence of freezing during cooling and cold crystallization upon reheating, reflecting constrained mobility arising from strong intermolecular interactions within the polymeric matrix. On the other hand, weakly bound water is defined by bulk-like freezing during cooling and vaporization at 100 °C, consistent with weak intermolecular interactions.

Under current frameworks, the mechanisms by which bulk and weakly bound water modulate polysaccharide chains are relatively well established. Marcombe et al. introduced equilibrium conditions for inhomogeneous swelling in hydrogels.^5^ Hong et al. introduced a thermodynamic framework for nonequilibrium in hydrogels assuming colloidal diffusion.^6^ Consistent with these frameworks, bulk water modulates polysaccharide chain mechanics through plasticization and swelling by disrupting the intermolecular networks.^7,8^ Here, we consider whether interfacial confinement of water introduces additional thermodynamic contributions that are not captured within these descriptions.

Recent studies, albeit not focused on polysaccharides, have highlighted the influence of interfacial water in material networks,^9,10^ yet these observations have largely been constrained to microscopic chemical and biological properties, and still lack a cohesive generalizable thermodynamic interpretation. For instance, the effect of bound water on the crystalline structure of chitosan films has been studied under varying levels of hydration.^11^ In another study, hexanoyl glycol and glycol substituents were introduced to chitosan, resulting in increased interfacial water content and altered interactions with blood platelets, thereby improving hemostatic biocompatibility.^12^ However, a generalizable thermodynamic framework describing how water is confined within interfacial polymer networks and how this confinement influences bulk properties remain absent.

This gap also limits the ability to advance the design of polysaccharide-based materials whose properties depend on controlled hydration states, despite the broad technological relevance of these systems in coatings,^13^ packaging,^14^ membranes,^15^ and biomedical interfaces.^16^ A mechanistic understanding of how water behaves when geometrically constrained along polysaccharide chains would therefore enable a more deliberate modulation of material properties.

Here, we introduce a thermodynamic framework in which mechanical softening arises from the confinement of interfacial water and the consequent generation of disjoining pressure. Under steric constraints, we find that water molecules organize into energetically unfavorable, low-entropy configurations. This water-driven mechanism is herein referred to as hydro-softening. Specifically, we demonstrate this using chitosan films processed on polydimethylsiloxane (PDMS), interfacially coupled with tetraethyl orthosilicate (TEOS). We find that substrate-induced changes in surface energetics produce measurable changes in both interfacial water signatures and the mechanical response of the chitosan films. Figure 1A illustrates the schematic of this material system; Figure 1B shows the interfacial linkage between chitosan, TEOS, and PDMS; and Figure 1C presents a close-up SEM image.

**Figure 1:**
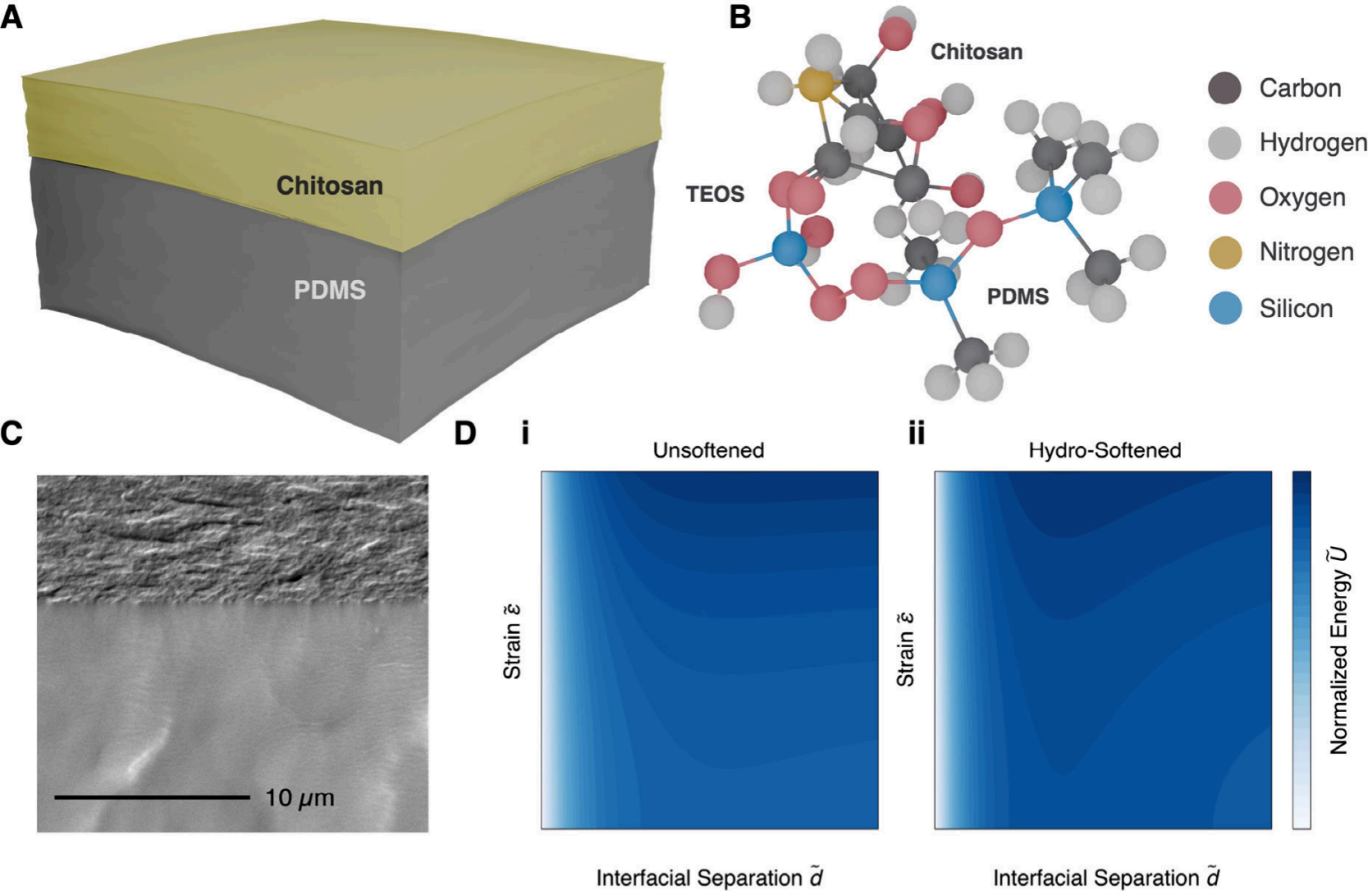
Schematic representation and associated analysis. (A) Schematic representation of chitosan thin film deposited on a PDMS substrate. (B) Molecular representation of the chitosan-TEOS-PDMS interface, illustrating interfacial connectivity. (C) Cross-sectional SEM image of the chitosan film on PDMS, showing the film–substrate interface. (D) (i) Free energy landscape as a function of normalized strain and interfacial separation, illustrating monotonic energy profile in untreated films. (D) (ii) Free energy landscape exhibits curvature arising from increased chain mobility induced by interfacial water under hydro-softened conditions.

## Results and Discussion

### Disjoining Pressure

Hydro-softening can be understood as a thermodynamic process in which water molecules, once confined within polymeric interfaces, are forced into an increasingly orderly state with reduced entropy. This confinement generates disjoining pressure, which drives adjacent polymer chains apart to restore energetic balance. We hypothesize that the resulting chain displacement manifests as enhanced mechanical compliance.

From the perspective of classical thermodynamics, the interfacial water framework is built upon two key theoretical constructs: Gibbs’s capillary theory^17^ and Derjaguin’s disjoining pressure theory.^18^ Gibbs’s capillary theory describes the balance of forces acting across confined polymer–water interfaces, including van der Waals, electrostatic, hydration, and entropic contributions.^19^ Derjaguin’s formulation validates this through the concept of disjoining pressure Π, which is defined as the derivative of the Gibbs free energy with respect to the interfacial separation distance:^20^

1 $$\Pi \left(d\right)=-{\left(\frac{\partial G}{\partial d}\right)}_{T,\mu }$$

where *G* is the Gibbs free energy, *d* is the interfacial distance, *T* is the temperature, and μ is the chemical potential. As Derjaguin’s formulation is defined as the derivative of the Gibbs free energy from, the equilibrium of interfacial pressure can be identified when *∂G*/*∂d* = 0, and thermodynamic stability can be identified when *∂* ^2^ *G*/*∂d* ^2^ > 0, at finite separation.

In the context of hydro-softening, we consider confined water a medium that induces disjoining pressure between polymer chains within the film, thereby elevating the free energy of the system. The thermodynamic penalty associated with this confinement is translated into reduced mechanical resistance, with softening emerging as the response to balance interfacial energetics. This framework can be further clarified through the Legendre transform of the Gibbs free energy:

2 G=U+pV−TS

where *pV* is the mechanical pressure–volume work term, and *TS* is the entropic term (product of temperature and entropy). Substituting the differential form of *U* from Equation 2 with respect to *G* makes explicit how interfacial confinement contributes to the thermodynamics of the system:

3 $$dU=-\Pi_{d}dd-SdT-n_{w}d\mu_{w}+\sigma d\varepsilon$$

where *S* is entropy, *n*_*w*_ is the number of water molecules, *μ*_*w*_ is the chemical potential of water, σ is stress, and *ε* is strain. These terms correspond to conventional conjugates: disjoining pressure versus distance, entropy versus temperature, chemical potential versus particle number, and stress versus strain.

As shown in eq 2, Gibbs free energy increases when the entropic term (*TS*) is reduced under confinement. And as we can understand from the differential form, eq 3, steric perturbations decrease with separation, *d*, thereby elevating the disjoining pressure term, Π_*d*_, shifting the chemical potential of confined water *n*_*w*_ *dμ*_*w*_, both of which contribute to a net increase in *G*. Accordingly, steric confinement reduces the entropy associated with water and elevates the Gibbs free energy of the system. To maintain equilibrium, this penalty is offset by a reduction in the elastic response *σdε*, whereby softening emerges as a compensatory mechanism that balances the interfacial energetics. This corresponds to weakening of cohesive interactions between polymer chains, effectively lowering the resistance to deformation. Thus, we find that steric effects emerge as a processing parameter capable of driving entropy changes in polymer–water configurations to induce hydro-softening.

To visualize how confinement and elasticity reshape the thermodynamic landscape under hydro-softening, we constructed a dimensionless phenomenological free-energy functional, *Ũ*(*d̃*, *ε*). The functional combines van der Waals cohesive attraction -1/*d̃*^2^, short-range disjoining pressure arising from interfacial water *ce* ^–*d̃*^, and a harmonic elastic term that sets the curvature along the strain axis $\frac{1}{2}k\varepsilon^{2}$ . Here, *c* and *k* are shape parameters that modulate the relative strength of confinement and elasticity. In the unsoftened state (Figure 1D (i), lower confinement (smaller *c*) and greater stiffness (larger *k*) generate a free-energy valley at modest separation with steep curvature along the strain axis *ε*, consistent with a stiff, cohesion-dominated geometry. Increasing the confinement parameter (*c*) and reducing the elastic penalty shifts the basin toward larger separation while flattening the strain direction, hydro-softening is characterized as a coupled thermodynamic-mechanical relaxation response (Figure 1D (ii). Disjoining pressure drives the chain apart, and the reduced elastic penalty enables the system to settle into a new low-energy state with both increased interfacial spacing and attenuated stiffness. The full derivation of the free-energy functional is provided in the Supporting Information, in the Free Energy Functional section.

### A Biologically Inspired Design

Biological systems have evolved diverse strategies to modulate mechanical compliance, exploiting interfacial water structuring as a functional design principle. But the extent to which water structuring affects mechanical compliance, and the mechanisms by which it operates vary distinctly across biological systems. In highly hydrated tissues (i.e., brain^21^ and mucosa^22^), mechanical softness arises from bulk water plasticization, where unbound and free water molecules increase conformational freedom of polymer networks. However, not all soft biological materials rely on overt wetness or plasticization to achieve compliance. In human skin, hydrogen bonding and steric crowding reorganize confined water within hydroxyl-rich glycosaminoglycan domains,^23,24^ generating local disjoining pressure without measurable volumetric expansion. The contrast between dry and hydrated skin reflects a shift in the hydration landscape. Similarly, dormant seeds remain mechanically rigid in a desiccated state until water infiltrates its amorphous polysaccharide- and protein-rich matrices in angstrom scale quantities.^25^ Within these confined domains, water reorganizes and generates pressure that counterintuitively reduces the fracture resistance, protecting the seed coats until germination.

With this understanding of disjoining pressure and its biological manifestations, we sought to reproduce a similar confined-water mechanism by mechanically imposing steric effects from the substrate during chitosan film processing and therefore eliciting the disjoining-pressure mechanism captured in the *d-σdε* relationship from eq 3. Our hypothesis is that a hardened substrate introduces steric constraints during chitosan film formation, driving the confinement of tightly bound water and effectively softening the chitosan film. This may seem counterintuitive, as it implies that soft films form on hard substrates, and harder films form on soft substrates. But a similar phenomenon with a different material system has previously documented;^26^ and with the thermodynamic framework introduced here, we attempt to clarify the role of interfacial water molecules as well as the underlying mechanism.

Chitosan was chosen as the model material system for this work, as its inherent stiffness reflects the highly crystalline organization of β-1,4-linked glucosamine structure and the dense network of interchain hydrogen bonding.^27^

### Steric Effects and Mechanical Softening

To control steric effects during hydro-softening, the substrates on which chitosan was processed were prepared with varying stiffness. PDMS substrates were prepared by thermal curing under controlled conditions at either room temperature or 120 °C, followed by UV–O_3_ treatment to modulate surface energetics. Chitosan films were subsequently deposited onto the substrate and underwent TEOS-assisted condensation at the interface. Supporting Information The thermal hardening of the substrates was evidenced by their elastic moduli increasing from 0.6 MPa at room temperature to 2 MPa at 120 °C, with a rapid rise between 80 and 120 °C (Figure 2A) (see Figure S1A-D for complete raw tensile data). Conversely, the empirically isolated elastic moduli of the chitosan films decreased sharply from 91.2 ± 0.2 MPa to 34.8 ± 0.03 MPa, asymptotically softening on stiffer substrates (Figure 2B) (see Figure S2A-D for complete raw tensile data). This reciprocal behavior demonstrates that steric accumulation in the substrate directly governs the hydration landscape of the film and therefore mechanical compliance. By reducing configurational freedom at the interface, steric constraints induce entropic contraction of confined water in the chitosan matrix, stabilizing hydration states, and minimizing free energy. The presence of water and the resulting hydration landscape are discussed in subsequent sections. Films processed at 120 °C are hereby referred to as hydro-softened chitosan, whereas those processed at room temperature are referred to as untreated chitosan.

**Figure 2:**
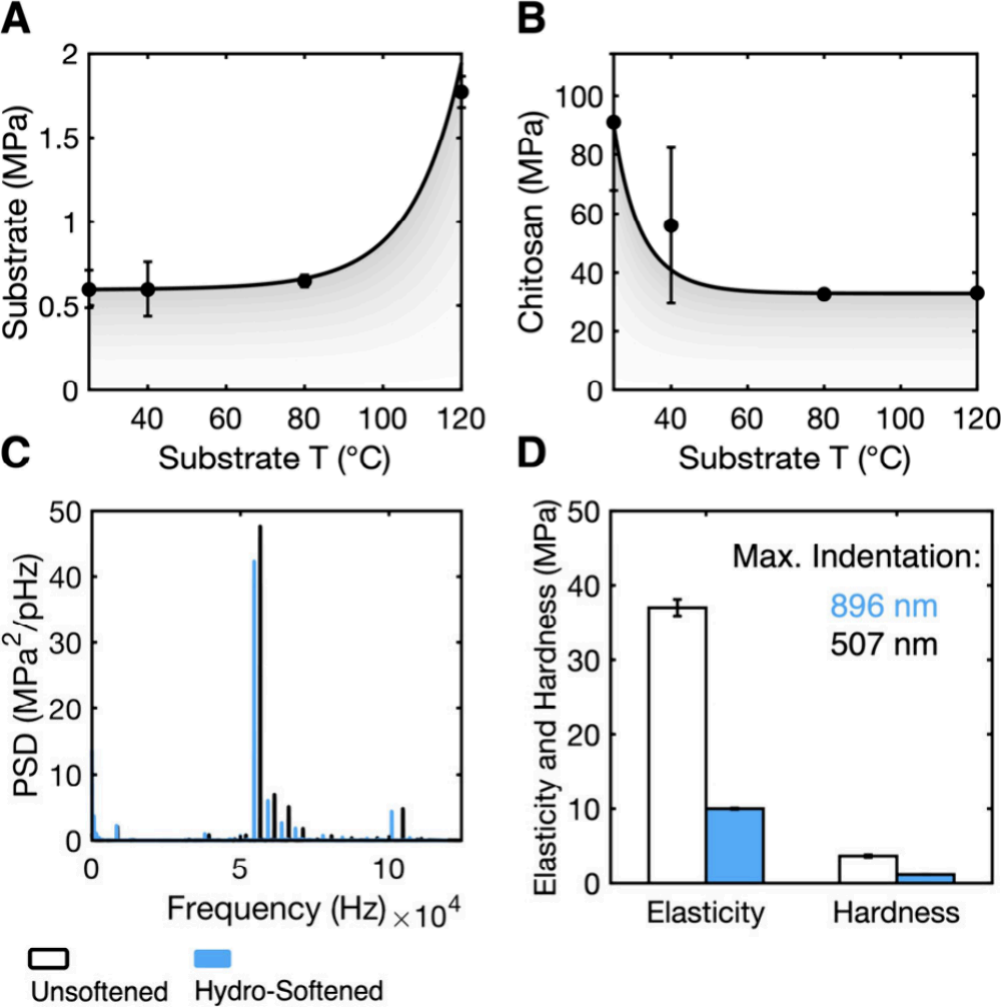
Mechanical response of hydro-softened versus unsoftened chitosan film. (A) Substrate elastic moduli increase with its processing temperature. (B) Isolated elastic moduli of chitosan film decreases as substrate temperature rises, reflecting softening induced by interfacial hydration. (C) Power spectral density (PSD) analysis reveals a marked reduction in high frequency mechanical fluctuations for hydro-softened films relative to unsoftened controls. (D) Nanoindentation confirms that hydro-softening lowers both elastic modulus and hardness, with increasing maximum indentation depth (896 nm vs 507 nm) when subject to an identical indent load, consistent with enhanced compliance from confined water.

Steric perturbations in the hydration landscape also manifest in the residual stress spectra of hydro-softened chitosan, as reported in Figure 2C. Power spectral density (PSD) analysis of the residual signal from tensile stress–strain curves reveals a marked leftward shift, corresponding to reduced high-frequency components. This attenuation beyond 5 × 10^4^ Hz indicates dampening of local mechanical fluctuations and enhanced molecular-level energy dissipation. The suppression of these fluctuations reflects a minimized Gibbs free energy and lowered disjoining pressure, thereby stabilizing the hydro-softened matrix and reducing variance in mechanical response.

Nanoindentations were performed with a controlled indent force to evaluate the softness of the hydro-softened and the unsoftened chitosan films, where hydro-softened chitosan exhibited a substantial reduction in both elastic modulus and hardness. As reported in Figure 2D, the elastic modulus decreased from 37.0 ± 1 MPa in unsoftened films to 11.4 ± 0.09 MPa in hydro-softened films, while hardness decreased from 3.73 ± 0.2 MPa to 1.14 ± 0.02 MPa. This reduction in mechanical resistance was accompanied by an increase in maximum indentation depth, from 507 to 896 nm, maintaining the same load during tests. See the Substrate Thickness Invariance section and the Tensile and Nanoindentation Analysis section of the Supporting Information for consistency analysis comparing tensile and nanoindentation measurements, based on Cauchy-Green deformation assumptions.

Together, our observations support that confined water lowers local stiffness and enhances polymer chain mobility. These mechanical signatures validate the role of hydration structuring in modulating nanoscale deformation behavior.

### Interfacial Water

The emergence of confined water is validated through experimental observations. Differential scanning calorimetry (DSC) measurements were used to reveal a distinct cold crystallization feature in hydro-softened chitosan, absent in the unsoftened chitosan, indicating the presence of water confined beyond typical freezing dynamics, as shown in Figure 3A. Cold crystallization is generally attributed to the reordering of tightly confined, nonfreezing water domains upon heating following prior suppression of crystallization upon cooling, and does not occur in loosely bound or unbound water due to the lack of cohesive structuring.^28^ This distinct behavior observed in hydro-softened chitosan aligns with the formation of sterically entrapped hydration domains.

**Figure 3:**
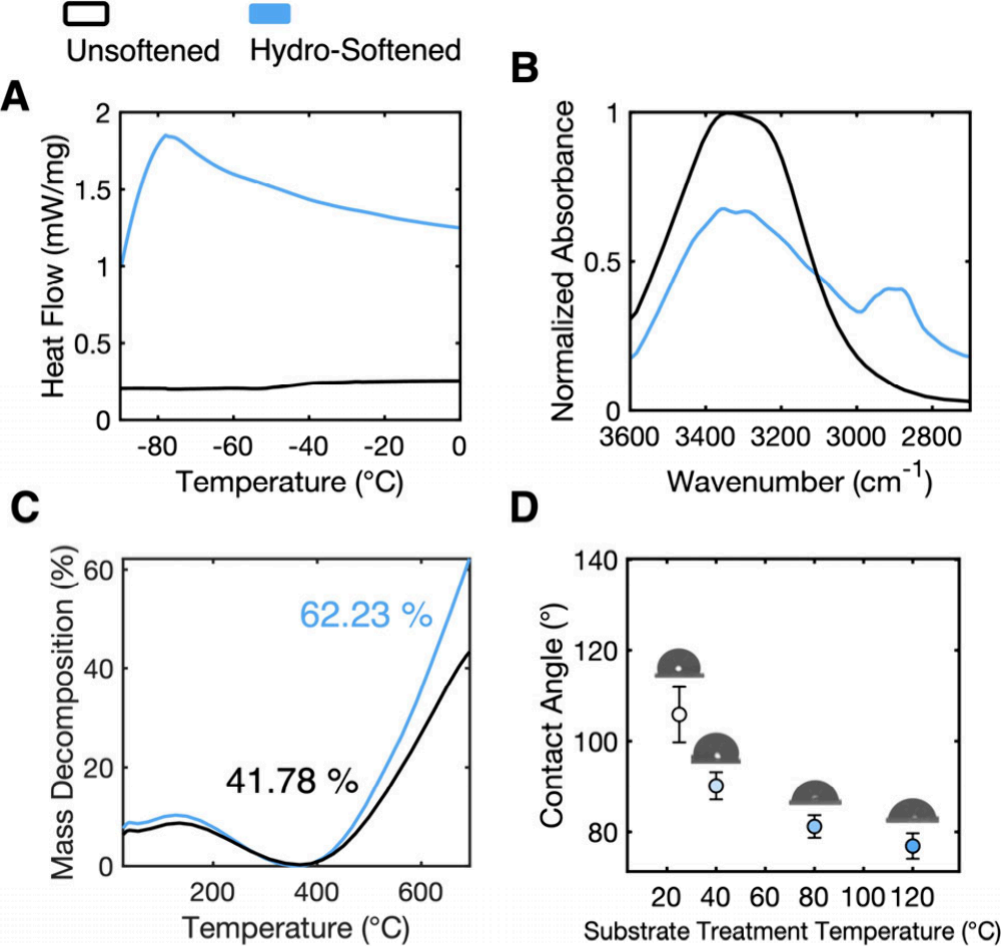
Thermophysical and spectroscopic evidence of hydro-softening. (A) Differential scanning calorimetry (DSC) shows a pronounced cold crystallization peak unique to hydro-softened conditions. (B) FTIR spectra reveal not only a redistribution of the broad O–H/N-H stretching band but also the emergence of an additional feature near 2900 cm^–1^, consistent with altered hydrogen bonding network within chitosan. (C) Thermogravimetric analysis (TGA) indicates mass decomposition above 100 °C, signifying stabilization by water beyond the unbound fraction. (D) Static water contact angle measurements of chitosan films on substrates processed at different temperatures.

Fourier transform infrared (FTIR) spectroscopy analysis further supports this interpretation, where hydro-softened chitosan exhibits a sharp absorbance peak at 2900 cm^–1^, in addition to the structured hydroxyl-water interactions at 3400 cm^–1^ ^29,30^ (Figure 3B, see Figure S3A-B for full FTIR spectra). The additional 2900 cm^–1^ peak suggests an increased ordering of water, consistent with tighter local confinement, as bound water in confined polymer matrices can shift OH stretching bands to lower wavenumbers due to an increase in hydrogen bond strengths.

As Persson and Halle have demonstrated, ^1^H nuclear magnetic resonance (NMR) is poorly suited for selectively probing interfacial water.^31^ In liquid NMR, the dominant bulk-water signal and rapid exchange dynamics mask the subtle relaxation differences of the confined fraction, thereby averaging interfacial contributions. In solid-state NMR, the population of tightly bound water is small and produces weak, broadened signals that overlap with the polymer backbone, while the interfacial fraction is neither fully solid nor fully liquid, yielding broad, averaged resonances with little selectivity. Although experimental NMR provides limited insight into confined hydration, density functional theory (DFT)-based NMR simulations conceptually support the idea that hydration perturbs the local electronic environment of protons in a predictable, site-specific manner. The resulting spectra in Figure S4 reveal a clear downfield shift in the hydrated chitosan model between 3.5 and 6.5 ppm, corresponding to protons adjacent to hydroxyls.

Thermogravimetric analyses (TGA) of hydro-softened and unsoftened chitosan from 100 to 600 °C uphold the presumed presence of bound water. To exclude the contributions from loosely bound water, which evaporates at temperatures below 100 °C, relative mass decompositions are analyzed over a temperature range of 100 to 600 °C. The relative mass loss in hydro-softened chitosan films measured at 62%, compared to the 42% (Figure 3C) in unsoftened chitosan films, suggesting that bound water persists beyond the initial drying stage and thermally degrades as the chitosan structure itself decomposes.

Beyond the presence of tightly confined water evident from thermal and spectroscopic signals, we further find that the balance of interfacial water states strongly influences the density of accessible polar groups, thereby shaping surface hydrophilicity. Changes in water contact angle linked to interfacial water have been well-documented; for instance, Cho et al. showed that introducing glycol and hexanoyl glycol groups into chitosan shifts the interfacial water states and yields distinct contact angles.^12^ A similar trend was observed with hydro-softening, with the contact angle reducing from 104° to 78° (Figure 3D). This behavior arises because steric confinement organizes interfacial water into structured, hydrogen-bonded domains, which amplify surface polarity and thereby enhance hydrophilicity, lowering the contact angle. Here, not only do we now detect clear signatures of interfacial water, but we also begin to establish that the interfacial water structuring is actively engaged in molecular-level rearrangements governing surface polarity and wettability. The molecular-level organization of these hydration clusters is further examined through simulations of the hydration landscape, providing insights into the geometric structuring of confined interfacial water in hydro-softened chitosan.

### Hydration Landscape

Here, we report a qualitative scalar field-based (see Figure S5 for simplified simulation architecture) interpretation of the chitosan-water interface to define a continuous hydration potential landscape generated by the superposition of atomic interactions within the amorphous domain of the polymer. We encode the spatial energetic favorability of water occupancy, classifying regions as unbound, weakly bound, or tightly bound, where migration of the hydration landscape is governed by the displacement of local energetic minima within the field, as steric conditions fluctuate. Tracking these shifts enables reconstruction of the evolving structure of confined water. With this framework, we elucidate that hydro-softening arises not merely from the presence of confined water, but from its entropically constrained organization within the hydrogen-bonded amorphous domains of chitosan. See the Hydration Weights section of the Supporting Information and Table S1 for details on how hydration weights are heuristically determined and encoded.

Simulation results reveal that steric confinement alters the hydration landscape around the chitosan chain upon hydro-softening. In the unsoftened state, hydration is diffusely distributed, with no preferential accumulation (Figure 4A (i). In contrast, the hydro-softened configuration exhibits anisotropic hydration clustering, particularly in regions sterically confined to the substrate interface (Figure 4B (i). These confined zones display locally elevated hydration values, consistent with a network orientation that favors the retention of high-energy water. This redistribution reflects a reduction in configurational entropy, effectively forcing water into constrained, less favorable states. To further resolve the spatial heterogeneity in hydration, we examined the cross sections of the simulation volumes (Figure 4A ii, B ii). These slices reveal a marked shift in the location and magnitude of maximal hydration between the unsoftened and hydro-softened configurations.

**Figure 4:**
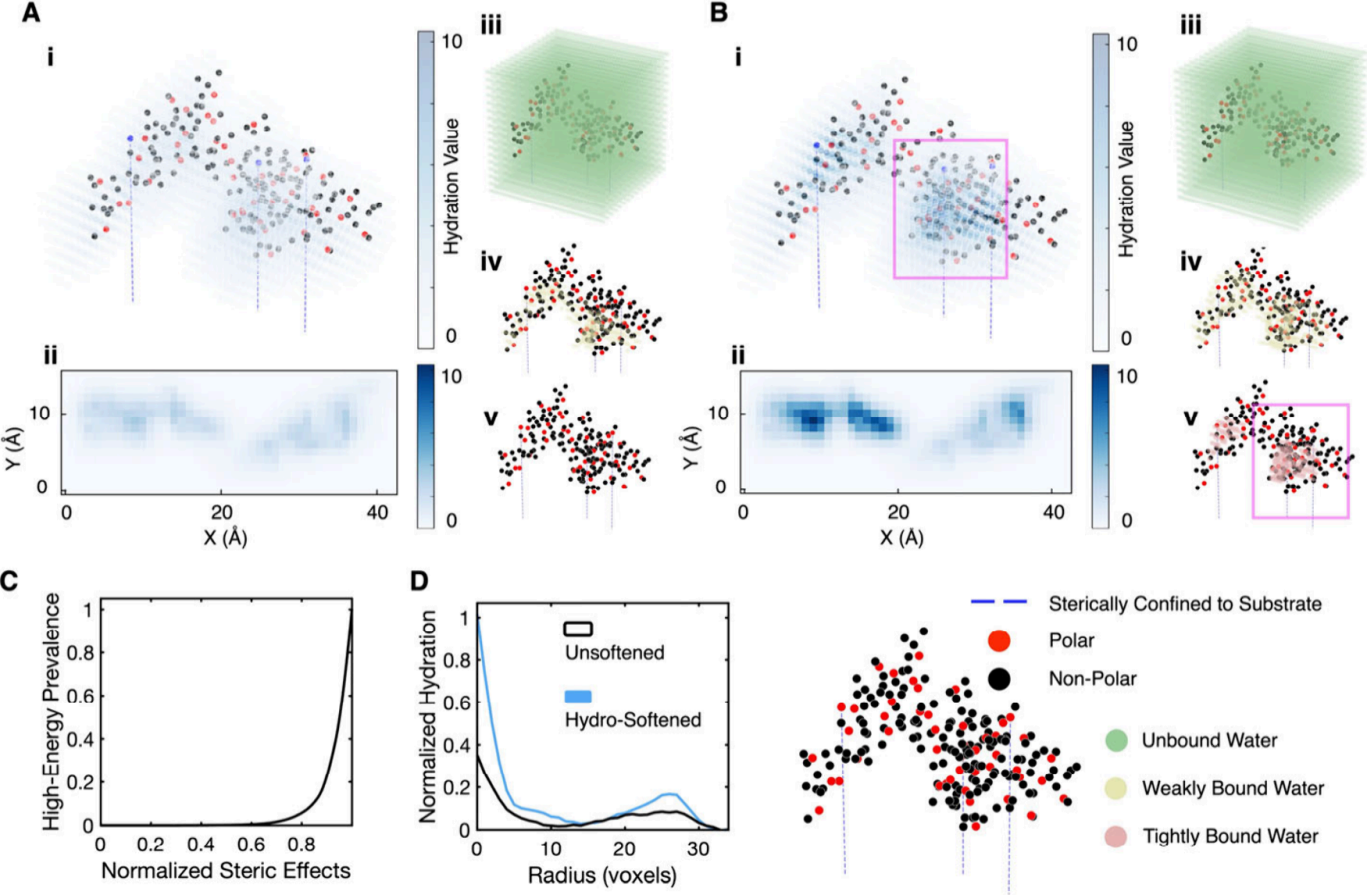
Physically informed simulations of interfacial hydration under (A) unsoftened and (B) hydro-softened conditions. (i) Three-dimensional hydration maps illustrating spatial interfacial water density around chitosan, with sterically confined regions indicated by dashed lines. (ii) Two-dimensional projections reveal intensified hydration near confined domains. (iii-v) Decomposition of hydration into unbound (green), weakly bound (yellow), and tightly bound (red) water mapped onto molecular frameworks. (C) High-energy water prevalence, showing a sharp increase with normalized steric effects. (D) Radial hydration profiles, showing reduced spread and elevated accumulated for the hydro-softened, indicating localized structuring of hydration.

The contrast is more apparent in comparing the distinct hydration regimes. Unsoftened chitosan primarily hosts unbound and weakly bound water (Figure 4A iii-iv), with minimal formation of tightly bound hydration shells as shown in Figure 4A v. In contrast, the hydro-softened chitosan exhibits a significant increase in tightly bound hydration clusters (Figure 4A iii-v), particularly in substrate-adjacent regions. This transition in hydration regime reflects how water molecules become transiently entrapped in constrained regions when unable to diffuse freely upon hydro-softening.

To quantify this emergent behavior, we calculated the prevalence of high-energy hydration states as a function of steric confinement. The probability density function of local hydration energy exhibits a pronounced nonlinear rise (Figure 4C), with high-energy prevalence remaining low under minimal confinement and increasing sharply as the steric effect increases. This trend is consistent with the anisotropy profiles (Figure S6A-B), where hydro-softened samples display steeper and more symmetric decay along all axes, reflecting enhanced structural confinement of water.

Radial decay profiles extracted from voxel-based hydration maps shown in Figure 4D also reveal a pronounced reduction in spatial spread, with hydro-softened samples exhibiting a tighter decay length (σ = 1.56 Å) relative to unsoftened films (σ = 1.79 Å), despite a significantly higher maxima in hydration potential. Furthermore, hydro-softened chitosan exhibits tighter and more symmetric directional decay profiles, indicative of entropically structured hydration. The Shannon entropy of the hydration field decreases from 2.1 in unsoftened films to 1.6 in hydro-softened films, reflecting a measurable loss in configurational disorder and confirming the emergence of spatially ordered, sterically stabilized hydration domains. The reduction in Shannon entropy quantitatively reflects the configurational loss predicted in eq 3, where steric perturbations elevate *G* by reducing the entropic term *S*. Thus, we find that the observed entropic contraction in the hydration field provides direct evidence of the thermodynamic penalty that drives mechanical softening.

## Discussion and Conclusion

The collective evidence elucidates a thermodynamic framework for confined water molecules in polysaccharides, demonstrating how interfacial hydration and disjoining pressure alter the mechanical response of the processed polysaccharide film. Specifically, we show that steric confinement arising from the substrate stiffness during thin film processing is responsible for the localization of hydration clusters, inducing entropic constraint throughout the polysaccharide chain. Subsequently, we show that the reduction in entropy imposed by water elevates the Gibbs free energy, and that this redistribution of Gibbs free energy results in a bulk mechanical change for chitosan films. However, the study is limited by the inability to resolve whether the softening originates from film-to-substrate interfacial confinement or from the central chitosan chains. And although the film softening reported in this work relies on substrate mediated steric modulations, the local entropy of interfacial water may be modulated in various ways, independent of the substrate energetics.

As some studies have shown that plasticization of chitosan increases the flexibility by limiting the formation of cohesive inter- and intramolecular hydrogen bonds in chitosan,^32^ the disruption of the physicochemical forces upon increased interfacial separation may also be relevant in a hydro-softened system. When the water is confined due to entropic effects, clusters of confined water could also be disrupting a cohesive network of hydrogen bonds, further relaxing the chain dynamics.

Ultimately, the resulting film is neither plasticized nor swollen, but instead represents a thermodynamically driven, water-mediated response. This mechanism preserves a level of compositional purity that is often compromised in more conventional methods of softening. In Figure 5, the compositional purity preserved by hydro-softening is contrasted against the softness-purity trade-off in conventional softening mechanisms (see the Calculation of Film Purity Trade-off section of the Supporting Information and Table S2 for complete data), where **Data point A** assumes water confinement to be localized to the film–substrate interface and **Data point B** assumes central chain confinement. **Data point C** assumes the nonfreezing water domain is confined between the central chain and the precursor. These data points reside well above the exponential regression boundary, providing clear evidence that hydro-softening overcomes the established purity-softness trade-off.

**Figure 5:**
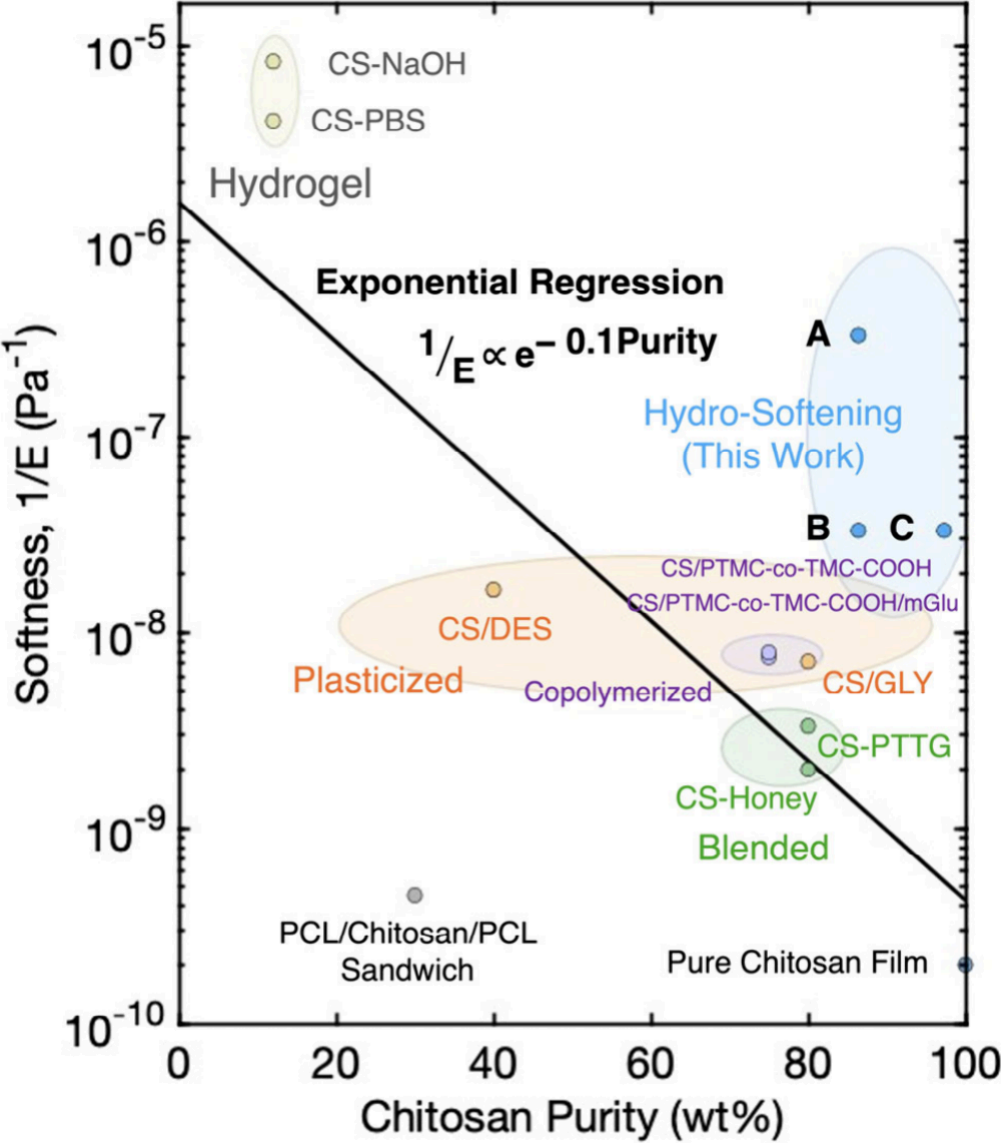
Softness-purity landscape of chitosan-based films. Softness (inverse effective modulus) is plotted against chitosan compositional purity. The solid line denotes an exponential regression boundary representing this softness-purity trade-off. Data point (A) assumes compositional purity when water is localized to film–substrate interface; (B) when confinement is within central polymer chains; and (C) when water is within the central chain and the precursor.

The mechanism reported in this work also largely affects substrate energetics, as reflected in the increasing hydrophilicity of chitosan films upon softening. The increasing hydrophilicity of the chitosan film surface as a result of softening, evidenced by decreasing contact angles, indicates a shift in the location of polar molecules on the chitosan surface, which implies disruptions in the migration of nonpolar polymers segments to the air–water interface during gradual drying.^33^ This phenomenon could be attributed to the thermally hardened substrate resulting in potential repulsion of polar molecules, disrupting the cohesive hydrogen bonds within the chitosan matrix.

Another point of interest concerns the nuanced role of water in this system. Hydration perturbs the native hydrogen bonding network within the polysaccharide matrix, yet the resulting water cluster remains highly entropically constrained. This structured hydration layer seems to be presenting a more polar interface to the contacting droplet on the film-to-air interface, increasing hydrophilicity, aligned with decreased contact angle.

Overall, the mechanism presented in this paper reduces the film stiffness by 63% (tensile) and 73% (nanoindentation) relative to the unsoftened controls. Moreover, in a recent study, we have reported that hydro-softening can alter the interactions between material interfaces and microbiome activities. The biological implications of hydro-softening^34^ invite a greater potential in elevating the development of flexible and wearable healthcare electronics, advanced barrier coating technology, and other biomedical applications.

Beyond the chitosan-PDMS system examined here, hydro-softening establishes a general pathway for engineering mechanically adaptive polymer interfaces without compromising chemical composition. This capability is particularly relevant for applications requiring simultaneous compliance and functional integrity, including soft robotics, skin-conformal biomedical devices, antimicrobial coatings, and flexible barrier films. In such systems, interfacial mechanics directly govern adhesion, transport, and biological interaction. The ability to modulate these properties through confined hydration, rather than bulk swelling or chemical modification, provides a scalable and chemically conservative strategy for designing responsive polymer systems across biomedical, packaging, and soft materials engineering contexts.

## Experimental Section

### Materials and Methods

Tetraethyl orthosilicate (TEOS), chitosan (MW 190,000–310 000 Da degree of deacetylation 75–85%), and hydrochloric acid (HCl) were purchased from MilliporeSigma. Sylgard 184 silicone elastomer kit (Dow Inc.) was purchased through Krayden Inc. To process the purchased powdered chitosan into film, first, 1 g of chitosan is protonated in a dilute acidic solution of 5.88 mL of 1 M HCl and 100 mL of deionized (DI) water. This is based on the stoichiometric ratio between available protons against chitosan amine groups:

1M HCl vol(mL)={1g×2×1000mL/L}/{[(12×12.01)+(24×1.008)+(2×14.01)+(9×16.00)}g/mol×1M]=5.88

The suspension is stirred at room temperature until fully homogeneous. To silanize this chitosan solution, 260 μL of TEOS is added to achieve a 40% degree of substitution following hydrolysis (Figure S7A-B):

$$\mathrm{TEOS vol}.\left(\mathrm{mL}\right)=\frac{1g\times 0.40\times \frac{208.33g/\mathrm{mol}}{340.33g/\mathrm{mol}}}{0.93g.\mathrm{mL}}=260\mu L$$

The mixture is stirred to ensure complete hydrolysis and precursor activation:

$$\mathrm{Si}{\left({\mathrm{OC}}_{2}H_{5}\right)}_{4}+4H_{2}O\to \mathrm{Si}{\left(\mathrm{OH}\right)}_{4}+4C_{2}H_{5}\mathrm{OH}$$

Polydimethylsiloxane (PDMS) substrates were prepared using the Sylgard 184 silicone elastomer kit by mixing the base and the curing agent at a 10:1 ratio by weight. The prepolymer was spin-coated onto cleaned silicon wafers (acetone, isopropanol, and DI water rinse) at 800 rpm for 60 s to yield a dry film thickness of 200 μm (Figure S8A). The substrate was cured at four different temperatures (room temperature, 40 °C, 80 °C, and 120 °C) to induce varying network densities and steric environments. Following thermal curing, PDMS substrates were treated in a UV–O_3_ chamber for 2 h to introduce surface hydroxyls (Figure S8B-D). The hydrolyzed chitosan was spin-coated onto the activated PDMS surfaces at 1200 rpm for 60 s, enabling condensation reactions between chitosan and PDMS hydroxyls at room temperature:

$$\begin{array}{l} \mathrm{Si}{\left(\mathrm{OH}\right)}_{4}+\mathrm{Si}{\left(\mathrm{OH}\right)}_{4}\to \mathrm{HO-}\mathrm{Si-}\mathrm{O-}\mathrm{Si-}\mathrm{OH}+H_{2}O,\\ \mathrm{Ch-}\mathrm{OH}+\mathrm{Si}{\left(\mathrm{OH}\right)}_{4}\to \mathrm{Ch-}\mathrm{O-}\mathrm{Si}{\left(\mathrm{OH}\right)}_{3}+H_{2}O,\\ \mathrm{Ch-}\mathrm{OH}+\mathrm{Si}{\left(\mathrm{OH}\right)}_{3}\left({\mathrm{OC}}_{2}H_{5}\right)\to \mathrm{Ch-}\mathrm{O-}\mathrm{Si}{\left(\mathrm{OH}\right)}_{3}+C_{2}H_{5}\mathrm{OH} \end{array}$$

Condensation progressed over 30 min. Here, interfacial steric effects dictate the extent of entropic confinement. Full schematic of this fabrication process is found in Figure S9. The resulting chitosan films exhibited uniform thickness and smooth surface morphology in cross-sectional SEM analysis, with compositional consistency confirmed by Energy-Dispersive X-ray Spectroscopy (EDS) (Figure S10A-B).

### Mechanical Tests

Tensile properties were measured using the Mark-10 ESM 303 with the films subject to a constant deformation rate of 1 mm min^–1^. The samples were cut into rectangular shapes with ratios following the American Society of Testing and Materials (ASTM) D3039/D3039 M Standards. The elastic modulus was determined from the slope of the stress–strain response within the initial 3% strain range, selected to ensure that the analysis remain within the linear-elastic regime of the material, a standard approach for characterizing soft polymeric materials. Tensile properties were analyzed using Voigt’s iso-strain model^35^ for layered laminates. The effective bilayer elastic modulus was decomposed into chitosan and PDMS contributions based on cross-sectional area fractions, allowing the isolated chitosan modulus to be calculated:

$$E_{\mathrm{Chitosan}}=\frac{\left[E_{\mathrm{Bilayer}}\left(A_{\mathrm{Chitosan}}+A_{\mathrm{PDMS}}\right)-E_{\mathrm{PDMS}}A_{\mathrm{PDMS}}\right]}{A_{\mathrm{Chitosan}}}$$

To assess local mechanical fluctuations, Fast Fourier Transform (FFT) was applied to the residual stress signal obtained after linear fitting of the 3% strain regime. The resulting power spectral density (PSD) was calculated using a Hann window and normalized against strain resolution to characterize frequency-dependent noise:

$$\mathrm{Var}=\frac{1}{N}\overset{N}{\underset{i=1}{\sum }}{\left(\sigma_{\mathrm{chitosan},i}-{\mathrm{\sigma ̂}}_{\mathrm{chitosan},i}\right)}^{2}$$

and σ̂_chitosan,i_ is the linear fit prediction.

To eliminate the coupling effect that may arise due to the substrate, nanoindentation tests were conducted solely on and within the chitosan layer to assess the softness and the elastic modulus of the hydro-softened and unsoftened chitosan films with a controlled indent force. The elasticity and hardness of unsoftened and hydro-softened chitosan films were measured using the Bruker Hysitron TI-980 Triboindenter with a 10-μm conospherical probe. A constant 25 μN load was applied to each film, with probing maximum depth controlled to be within the thickness of the chitosan film (10 μN). The linear-elastic regions of each sample were also confirmed applying an incremental load across 1 μN to 100 μN. Data were collected using both single-test and 3 × 3 high-speed property mapping (XPM) with a standard quasi-static (QS) nanoindentation, and the results were averaged for each sample. Defining the contact radius as

r=Aπ

the r/t_chitosan_ for hydro-softened chitosan and unsoftened were 0.25 and 0.15 respectively, consistent with reported protocols for isolating substrate compliance effects.^36^

### SEM

Surface and cross-sectional morphologies were examined using a Phenom XL G2 scanning electron microscope (SEM) operating at 10 kV. Prior to imaging, samples were immersed in a 70% v/v ethanol for 15 min and dehydrated at 40 °C to minimize charging artifacts and to improve imaging stability. Cross-sectional views were prepared by freeze-fracturing the samples in liquid nitrogen. All samples were sputter-coated with a thin layer of gold for 45 s using a Cressington 108 sputter coater to enhance conductivity during SEM observation. EDS spectra were acquired with the Phenom ProX G5 system to validate the elemental distribution.

### Thermal Signals

Differential scanning calorimetry (DSC) was performed on the hydro-softened and unsoftened samples using a Mettler Toledo DSC3+ under a nitrogen purge (50 mL min^–1^). Approximately 5 to 10 mg of each sample was hermetically sealed in standard aluminum pans. Data was collected in a double-loop measurement, with an isothermal equilibration at −90 °C for 8 min, followed by a heating ramp from −90 to 80 °C at 20 °C min^–1^. This cycle was repeated twice to ensure reproducibility and to differentiate reversible and irreversible transitions. Thermal signals in subzero temperature were analyzed to identify cold crystallization and quantify tightly bound water fractions. Thermogravimetric analysis (TGA) was conducted using a 20 °C min^–1^ linear ramp from 25 to 700 °C under nitrogen atmosphere. Here, weight loss below 100 °C was attributed primarily to the vaporization of loosely bound water, while the region above 100 °C was further analyzed to reflect the loss of the tightly bound water regime and the onset of polymer degradation.

### FTIR

FTIR spectra were collected using a Nicolet iS50 spectrometer in transmission mode. Each sample was scanned 32 times over the range of 4000 to 800 cm^–1^ at a resolution of 4 cm_–1_. Films were cast onto IR-transparent substrates and dried under ambient conditions prior to measurement to minimize free water interference. Spectra were normalized to their respective baselines, and O–H stretching (2900 to 3600 cm^–1^) was analyzed for hydration-related features.

### Contact Angle

Static contact angles were measured with a Rame-Hart goniometer (Model 250) and DROPimage Advanced software. A 4 μL droplet of DI water was deposited on the coated chitosan film using the sessile drop method, and the angles at the droplet edges were recorded. The angle was measured at 30 s after the water drop. Each measurement was recorded by taking the average of at least three runs.

### DFT

To evaluate local electronic environment of protons in chitosan and its hydrated analogue, DFT-based ^1^H NMR simulations were carried out with the PySCF package with the 6–31G­(d) basis set. Gaussian simulation results were used to define the hydration shells around chitosan: approximate radius (1.25 Å) of the water cluster, corresponding to a 2.5 Å diameter sphere. Then, we randomly seeded points inside the sphere and used K-Means clustering to collapse them into representative hydration geometries, with each cluster centroid defining a distinct arrangement of waters around the polymer. We carried out a B3LYP/6–31G­(d) Kohn–Sham DFT optimization (RKS module)^37,38^ for each snapshot and ran a GIAO NMR calculation^39^ on the converged wave functions. We then extracted the isotropic average from the H shielding tensors, converted it to a chemical shift relative to tetra-methyl-silane, and plotted the results on the stick spectrum.

### Simulation

The Gaussian simulation reported in this study returns an energy field from hydration-specific spatial and directional information inaccessible to trajectory-based molecular dynamics simulations.^40^ The simulation is a voxel-resolved quantification of hydration probability as a function of atomic identity, directional preference, and steric shielding. Atomistic coordinates, G­(*x*, *y*, *z*), representing the spatial hydration probability distributions, can be modeled as

$$G\left(x,y,z\right)=\frac{1}{{\left(2{\pi \sigma }^{2}\right)}^{3/2}}\exp\left(-\frac{x^{2}+y^{2}+z^{2}}{2\sigma^{2}}\right)$$

where *x*, *y*, *z* are the Cartesian coordinates of a point in space relative to the reference atom, and σ is the standard deviation of the Gaussian kernel, representing the spatial decay length of hydration probability. The use of σ follows the conventional notation for standard deviation and should not be confused with mechanical stress. The kernel is normalized to unit volume and forms the spatial component of a directionally biased hydration model, with anisotropy introduced through atom-specific preferred vectors and angular weighting. The hydration weights were heuristically assigned to atom types to approximate their relative affinity for water, informed by their respective Pauling electronegativity and qualitative hydration enthalpy trends.

In encoding the effect of steric accumulation in the energy field, we define retained water volume, *V*_*w*_, to be proportional to the weighted sum of local hydration potentials, modulated both by steric proximity and the cumulative shielding effect:

$$V_{w}\propto \underset{i,j,k}{\sum }H\left(i,j,k\right)\exp\left(-\frac{d_{i,j,k}^{2}}{2\sigma^{2}}\right)$$

where *H*(*i*, *j*, *k*) is the hydration field intensity at a given voxel (*i*, *j*, *k*) and *d*_*i*__, *j*, *k*_ is the distance to the sterically active center.

To quantify the entropic effects of steric confinement on hydration energetics, we computed the prevalence of high-energy hydration states as a function of steric influence. This relationship was derived from voxel-based hydration energy distributions obtained under varying degrees of steric constraint. For each steric condition ϕ, we calculated the cumulative probability of hydration energy exceeding a threshold *ψ*_*thresh*_ by integrating the tail of the distribution:

$$P_{high-energy}\left(\phi \right)=\int_{\psi_{thresh}}^{\infty }\rho \left(\psi ;\phi \right)d\psi$$

where ρ­(ψ; ϕ) is the probability density function of local hydration energy ψ under steric influence ϕ, capturing how increasing steric influence reduces the local configurational entropy of water, thereby increasing the statistical likelihood of high-energy hydration states.

## Supplementary Material



## Data Availability

The data supporting the findings of this study are available within the paper and its Supporting Information files. In compliance with institutional data management policies, the full codebase for the simulation reported in this study is maintained on Georgia Tech’s GitHub Enterprise instance. A stable public version is available at https://github.com/hseo47/AQUA and has been archived with a permanent DOI at 10.5281/zenodo.15815360. This public version includes usage instructions, example data sets, and all necessary dependencies for replication.

## Notes

Free energy functional; substrate thickness invariance; kinetically hindered hydrophobic recovery under thermal processing; tensile and nanoindentation analysis; molecular dynamics; hydration weights; calculation of film purity trade-off; Figures S1–S12; Tables S1–S5; Supporting Information references (PDF)
