# Supplementary material for: Thermodynamics of Interfacial Water Confinement Reveal Altered Mechanical Properties in Polysaccharides Films
Source: ACS Polym Au. 2026 Mar 16;6(4):1062–71. doi: 10.1021/acspolymersau.6c00003 (PMC13474351; doi:10.1021/acspolymersau.6c00003)
Supplement: Supplementary file 1 [file lg6c00003_si_001.pdf]

Thermodynamics of Interfacial Water Confinement Reveal Altered Mechanical Properties in Polysaccharides Films

*Hojin Seo†, Xiaoqing Yu†, and Tequila A. L. Harris**

George W. Woodruff School of Mechanical Engineering,

Georgia Institute of Technology, Atlanta, GA, 30332-0405, USA

†These authors contributed equally

*E-mail: tharris3@gatech.edu

Supporting Information

FREE ENERGY FUNCTIONAL. To visualize the qualitative effects of confinement induced disjoining pressure and elastic compliance on the thermodynamic landscape, we construct a simplified, dimensionless phenomenological free-energy functional $\tilde{U}(\tilde{d}, \varepsilon)$ expressed in terms of interfacial separation $\tilde{d}$ and strain ε. The functional form is chosen to represent the lowest-order contributions expected to govern stability and relaxation in confined hydration system in polymer, while remaining analytically simple and free of material-specific parameters. The free energy is expressed as:

$$\tilde{U}(\tilde{d}, \varepsilon) - \frac{1}{\tilde{d}^2} + ce^{-\tilde{d}} + \frac{1}{2}k\varepsilon^2.$$

The first term, $-\frac{1}{\tilde{d}^2}$, represents a cohesive attraction between polymer chains. Power-law attractive interactions are widely used in continuum and interfacial theories as coarse-grained representations of van der Waals type cohesion to ensure that, in the absence of strong repulsive contributions, the system exhibits a stable minimum at finite separation[1]. The precise exponent is not intended to be unique; rather, it is selected to yield a smoothly decaying attractive contribution that dominates at moderate separation and provides a baseline cohesive tendency.

The second term, $ce^{-\tilde{d}}$, is introduced to represent a short-range repulsive interaction associated with confinement-induced ordering of interfacial water. Exponentially decaying repulsive forces are a feature of Derjaguin-type disjoining pressure formulations and hydration force models, reflecting the rapid decay of confinement and structural ordering effects with distance[2]. The dimensionless coefficient c modulates the relative magnitude of this confinement penalty and is used to present changes in the degree of water confinement.

The third term, $\frac{1}{2}k\varepsilon^2$, is a harmonic elastic contribution that penalizes deformation. A quadratic dependence on strain corresponds to the lowest-order term in a Taylor expansion of elastic energy and is standard in continuum descriptions of small deformations[3]. This term sets the curvature of the free-energy surface along the strain coordinate and provides a simple means of representing differences in mechanical stiffness. The dimensionless parameter k controls this curvature and is used to distinguish between mechanically stiff and compliant domains.

SUBSTRATE THICKNESS INVARIANCE. The measured thickness for each PDMS curing condition is shown in Table S3. To demonstrate thickness changes across various substrate curing conditions, we performed volume-controlled drop-casting in glass petri dishes. The casting volume of PDMS mixtures was controlled at 5 mL, and the mixtures were cured under various thermal conditions. After the PDMS substrates were fully cured, the thickness of each film was measured and recorded. A 2 hr UV-O3 treatment was performed prior to another 5 mL drop casting of the chitosan solution. The films were left under ambient conditions for 24 hours (24 ˚C, 44-45% humidity) to induce condensation. We then measured the dry thickness of each film and documented it in Table S3. The PDMS substrate showed a subtle increase in thickness with increasing curing temperatures. However, the chitosan film thickness, measured through subtraction was invariant to the substrate processing temperatures. Chitosan films cast on the 120 ˚C cured substrate were slightly thinner, and obtained lower elastic modulus compared to chitosan films cast on the RT cured substrate. The chitosan thickness invariance (when coupled with observed cold crystallization of interfacial water, detailed in the main text) leads the authors to believe that the reduction in elastic modulus is unlikely to be driven by macroscopic swelling/plasticization. Instead, the steric effect induced by the thermally hardened substrate likely alters film formation and its microstructure, allowing confinement of interfacial water, governing the softening.

KINETICALLY HINDERED HYDROPHOBIC RECOVERY UNDER THERMAL PROCESSING. Surface energetics of PDMS vary with curing temperature. This variation is critical for resolving hydration clustering within chitosan films processed on PDMS, as interfacial energy governs both water uptake and localized confinement within the polysaccharide matrix. Consequently, the temporal evolution of surface wettability provides a direct probe of the interfacial boundary conditions that regulate hydration behavior. To quantify this effect, UV-O_3-treated PDMS substrates cured at room temperature and at 120 ˚C were characterized via time-resolved water contact angle measurements. The contact angle was recorded at 1, 3, 5, 7, 9, 11, 15, 20, 30, and 60 minutes following droplet deposition (Table S4). Identical measurements performed on untreated PDMS substrates confirmed their hydrophobic nature and limited wetting (Table S5). The temporal evolution of the contact angle was modelled using a first-order kinetic framework of the form

$$\theta(t) = \theta_\infty - (\theta_\infty - \theta_0)\exp(-kt)$$

where θ_0 is the initial contact angle immediately after treatment, θ_∞ is the asymptotic contact angle at long times, and k is the effective rate constant governing surface recovery (Figure S11).

This formulation describes hydrophobic recovery as an exponential relaxation toward equilibrium and provides a reduced representation of interfacial reconstruction dynamics. First-order kinetic descriptions are widely employed in interfacial systems where a dominant relaxation pathway governs the evolution of an observable quantity. In this context, the model serves as an effective parameterization rather than a mechanistic decomposition, enabling the extraction of kinetic coefficients that reflect the accessibility of surface reconfiguration pathways. Comparable exponential formalisms have been used to describe hydration-mediated interfacial transitions, including molecular-scale hydrophobic interactions, where transitions between solvent states follow first-order kinetics[4]. Accordingly, this framework provides a physically consistent basis for quantifying hydrophobic recovery in PDMS.

Fitting of the experimental data reveals that PDMS cured at room temperature exhibits a significantly larger recovery rate constant ($k = 0.2618\ min^{-1}$), whereas PDMS cured at 120 ˚C shows a markedly reduced rate ($k = 0.0079\ min^{-1}$). Notably, the thermally processed substrate approaches a higher asymptotic contact angle ($\theta_\infty \approx 86.9^\circ$) compared to the room-temperature substrate ($\theta_\infty \approx 61.9^\circ$). This behavior indicates a decoupling between thermodynamic driving force and kinetic accessibility: despite a larger deviation from equilibrium, the 120 ˚C substrate recovers more slowly.

This decoupling is consistent with the presence of steric constraints at the interface. Thermal processing induces a structurally constrained near-surface region in PDMS, reducing configurational accessibility for chain reorientation and limiting the exposure of hydrophobic moieties. As a result, the rate of surface reconstruction is suppressed despite a larger thermodynamic gradient. These findings support the interpretation that steric effects, arising from discrepant dimensionality between the bulk PDMS network and the interfacial region, govern the dynamics of hydrophobic recovery and, by extension, the interfacial conditions that regulate hydration clustering within the chitosan matrix.

TENSILE AND NANOINDENTATION ANALYSIS. To reconcile the discrepancy in tensile and nanoindentation measurements, we applied the generalized Hookean framework for isotropic linear-elastic solids, where stress and strain are related by Cauchy-Green stiffness (C_{ijkl}) and compliance tensors (S_{ijkl}):

$$\sigma_{i,j} = C_{ijkl}\varepsilon_{kl},\ \varepsilon_{ij} = S_{ijkl}\sigma_{kl},$$

In matrix notation, the stiffness and the compliance tensors reduce to:

$$[C]=\begin{bmatrix} \lambda+2\mu & \lambda & \lambda & 0 & 0 & 0 \\ \lambda & \lambda+2\mu & \lambda & 0 & 0 & 0 \\ \lambda & \lambda & \lambda+2\mu & 0 & 0 & 0 \\ 0 & 0 & 0 & \mu & 0 & 0 \\ 0 & 0 & 0 & 0 & \mu & 0 \\ 0 & 0 & 0 & 0 & 0 & \mu \end{bmatrix} \text{ and}$$

$$[S]=\begin{bmatrix} \frac{1}{E} & -\frac{\nu}{E} & -\frac{\nu}{E} & 0 & 0 & 0 \\ -\frac{\nu}{E} & \frac{1}{E} & -\frac{\nu}{E} & 0 & 0 & 0 \\ -\frac{\nu}{E} & -\frac{\nu}{E} & \frac{1}{E} & 0 & 0 & 0 \\ 0 & 0 & 0 & \frac{1}{2G} & 0 & 0 \\ 0 & 0 & 0 & 0 & \frac{1}{2G} & 0 \\ 0 & 0 & 0 & 0 & 0 & \frac{1}{2G} \end{bmatrix}.$$

Here, the standard notations E and ν are the elastic modulus and the Poisson's ratio, respectively. λ and μ are Lamé parameters ($\lambda=\frac{E\nu}{(1+\nu)(1-2\nu)}$, $\mu=G=\frac{E}{2(1+\nu)}$). Under plane-strain uniaxial conditions ($\varepsilon_{yy}=\varepsilon_{zz}=\gamma_{xy}=0$), the effective elastic modulus simplifies to:

$$E_{Tensile}=E\frac{1-\nu}{(1+\nu)(1-2\nu)},$$

This factor serves as the plane-strain multiplier, which bridges the uniaxial tensile modulus with the triaxial constraint experienced during nanoindentation. For a representative Poisson's ratio range of 0.30–0.45, the plane-strain multiplier spans 1.35–3.80. The experimentally observed discrepancy reports a multiplier value of 2.45 for unsoftened chitosan and 3.09 for hydro-softened chitosan, thus quantitatively consistent with the theoretical multiplier.

MOLECULAR DYNAMICS. Prior to evaluating the thermodynamic response to the environmental changes that conditions hydro-softening, a molecular dynamics (MD) simulation[5] was performed using a chitosan 14-mer embedded in a Transferable Intermolecular Potential with 3-Points (TIP3P) water model[6]. The TIP3P water model was selected for its compatibility with Open Force Field Initiative (OpenFF) 2.0.0 and its reliable treatment of long-range electrostatic interactions. OpenFF 2.0.0 was selected for its adaptability in capturing conformational energetics, incorporating both bonded and non-bonded terms[7, 8]. The molecular system was constructed using Modeller class, with solvation and parameterization of chitosan and water handled independently to preserve modularity between

components. This MD simulation served to establish a structural and thermodynamic baseline of the hydration behavior of chitosan in the absence of steric confinement, enabling subsequent quantification of deviations from this baseline under modified environmental conditions (Figure S12A). Trajectory visualization for qualitative inspection was performed using Visual Molecular Dynamics (VMD)[9]. The system was energy-minimized and equilibrated prior to analysis. From the resulting trajectory, we extracted the root-mean-square deviation (RMSD) of the chitosan chain (Figure S12B) as well as the radial distribution function (RDF) of water oxygen atoms around chitosan heavy atoms (Figure S12C). While these trajectory-derived observables provide a useful baseline, they remain inherently limited for evaluating hydro-softening. Conventional MD trajectories primarily capture dynamic fluctuations and average structural properties, but they do not fully resolve the spatially heterogeneous, voxel-level energetics of confined hydration domains. The RMSD reflects conformational drift of the polymer backbone, and the RDF indicates average radial water distribution, yet neither captures the anisotropy, entropic contraction, or local energy penalties that define sterically confined hydration. For this reason, we employed a voxel-resolved statistical framework to quantify hydration probability fields and high-energy prevalence under steric perturbation, enabling a thermodynamically grounded interpretation of hydro-softening beyond the reach of conventional trajectory analysis.

HYDRATION WEIGHTS. Hydration weights were assigned by mapping atomic properties to a dimensionless scale that reflects the relative affinity of each atom type for water, using oxygen as a reference (weight%=%1.0). Two physicochemical properties guided this mapping: Pauling electronegativity (EN) and qualitative hydration enthalpy (ΔH_{hyd}). Oxygen (O), with the highest EN among the selected atoms (3.44) and a typical hydration enthalpy of approximately -400%kJ/mol, was selected as the baseline of 1.0. Nitrogen (N), slightly less electronegative (EN%=%3.04) but still capable

of strong hydrogen bonding, has a hydration enthalpy around -300%kJ/mol, and was assigned a weight of 1.4. In contrast, carbon (EN%=%2.55, ΔH_{hyd} =% − 80%kJ/mol) lacks the polarity or hydrogen bonding potential of O or N, and was therefore assigned a significantly lower weight of 0.3. Hydrogen (EN%=%2.20, ΔH_{hyd} =% − 20%kJ/mol) plays only an indirect role in hydration through donor bonding when covalently linked to electronegative atoms; in isolation, it exhibits very weak water affinity, justifying a minimal weight of 0.1. This heuristic scaling captures known solvation behavior and maintained numerical stability and tractability in the simulation study.

CALCULATION OF FILM PURITY TRADE-OFF. Chitosan purity was evaluated using two complementary approaches, reflecting different levels of system definition. At the film-on-substrate scale (PDMS-TEOS-chitosan), the average molar mass of the repeat unit was calculated as a function of the degree of substitution (DS), incorporating contributions from PDMS and TEOS linkers. For DS = 0.40, this yielded an average repeat unit of 394.023 $gmol^{-1}$, corresponding to a chitosan mass fraction of 86.4%. In contrast, when purity was defined for the film-only case (chitosan activated with TEOS precursor), we estimated the retained TEOS mass following hydrolysis-condensation, the reacted share (7%), and the substituted fraction (40%). Normalizing the reacted mass against the total film weight gave a chitosan content of 97.3%. These values were reported separately to capture both system-level and film-level perspectives on chitosan purity.

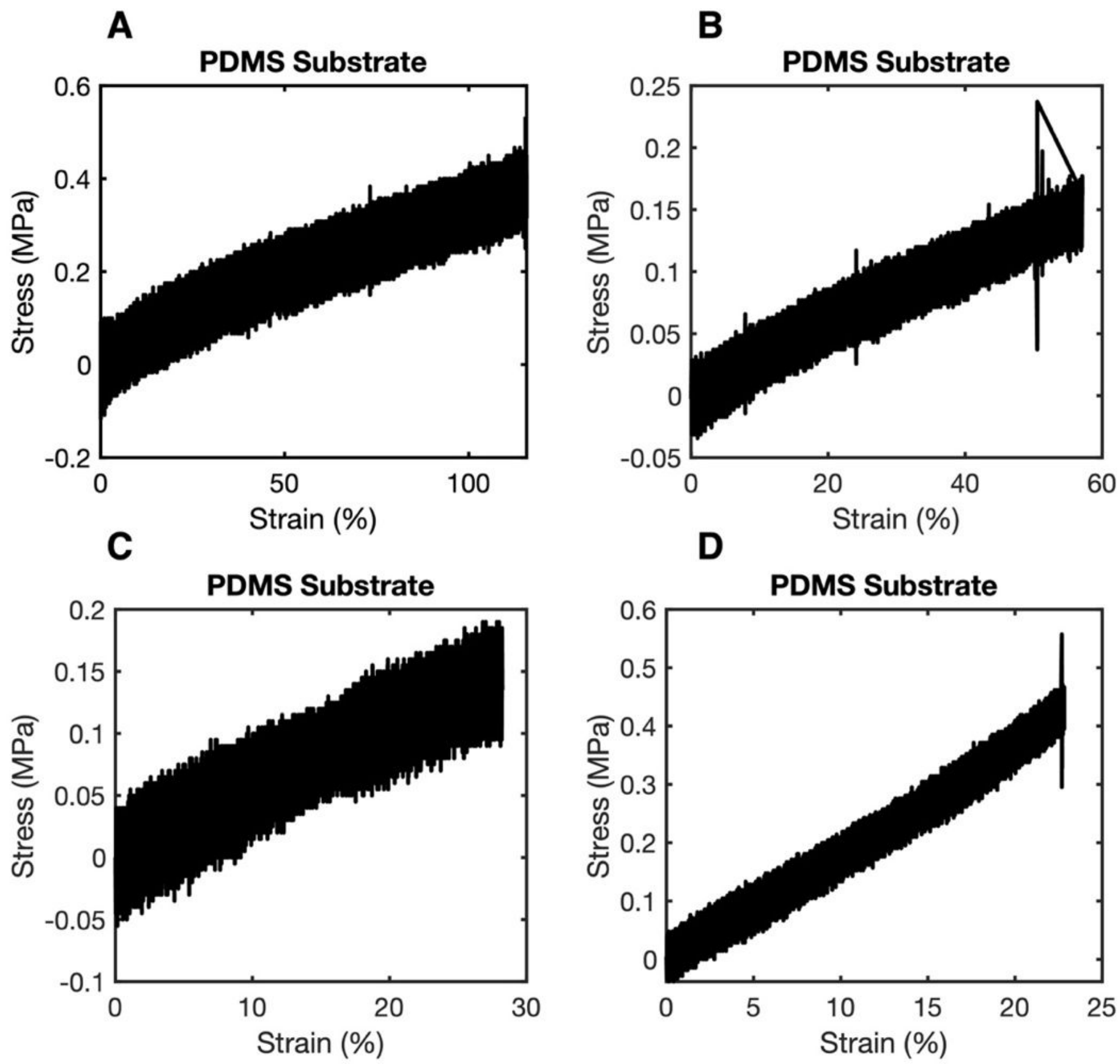

Figure S1. Tensile test results of the PDMS substrate. Tensile results of the PDMS substrate, cured at the following temperatures: **(A)** room-temperature **(B)** 40 °C **(C)** 80 °C **(D)** 120 °C.

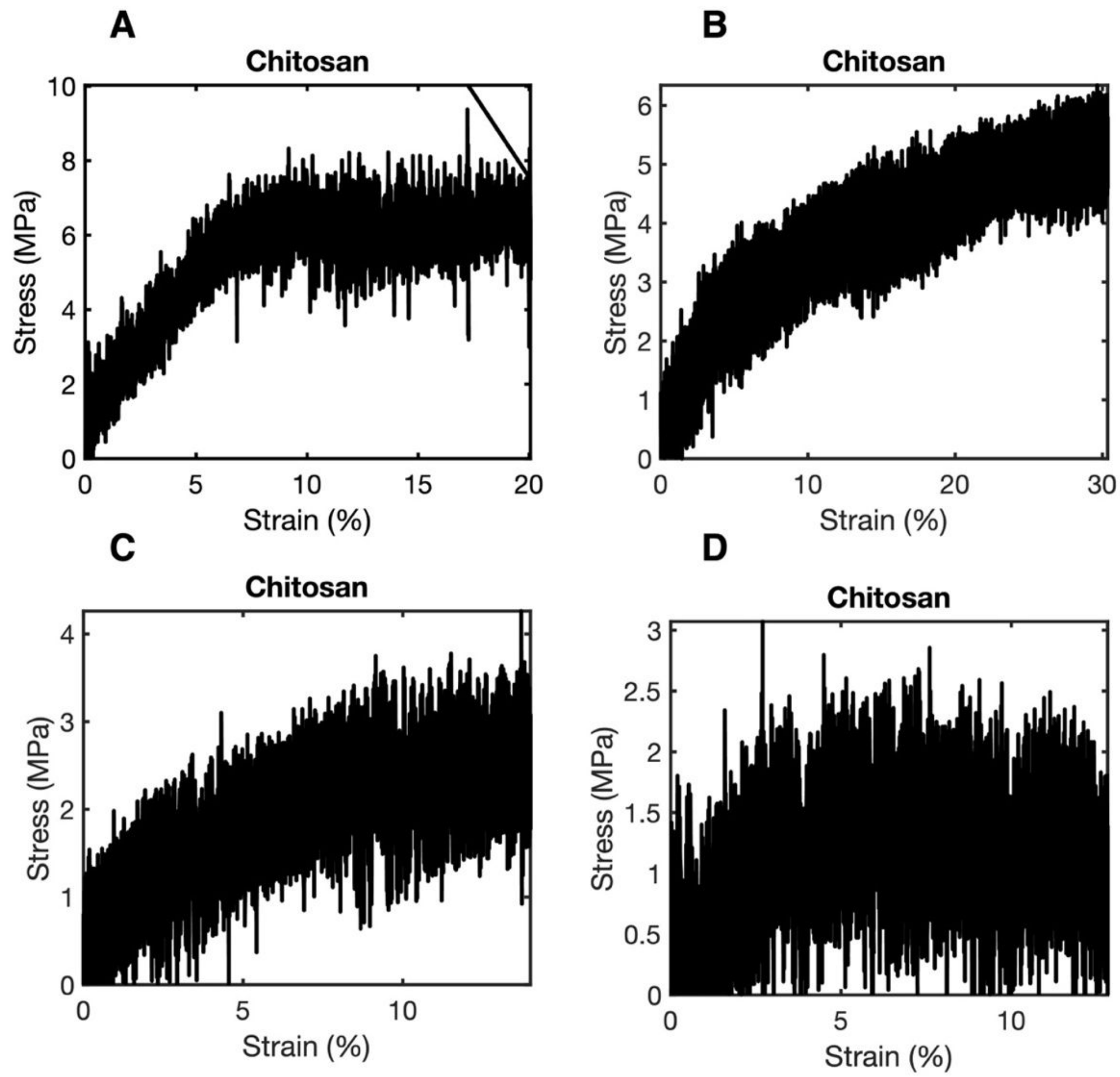

Figure S2. Chitosan tensile test results. Tensile results of the chitosan films, processed on PDMS substrates cured at the following temperatures respectively: **(A)** room-temperature **(B)** 40 °C **(C)** 80 °C **(D)** 120 °C.

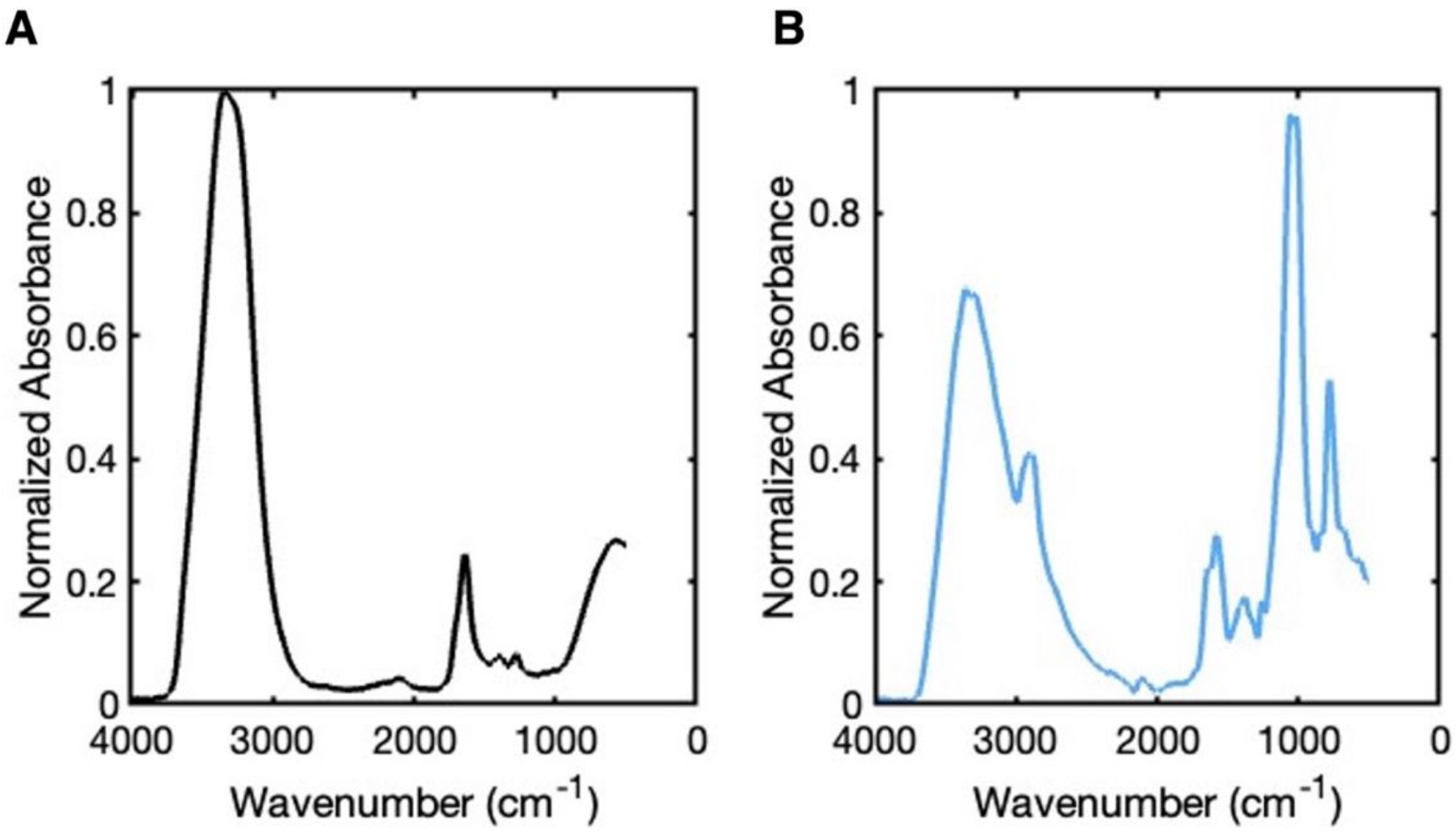

Figure S3. Full FTIR spectra for unsoftened and hydro-softened chitosan. Additional peak at 2900 cm^{-1} is indicative of hydroxyls in a tightly confined arrangement. Unlike unsoftened chitosan **(A)**, hydro-softened chitosan **(B)** also shows a more prominent H-O-H bending near 1650 cm^{-1}, and more pronounced peaks in the C-O stretching 1000 to 1200 cm^{-1} regions.

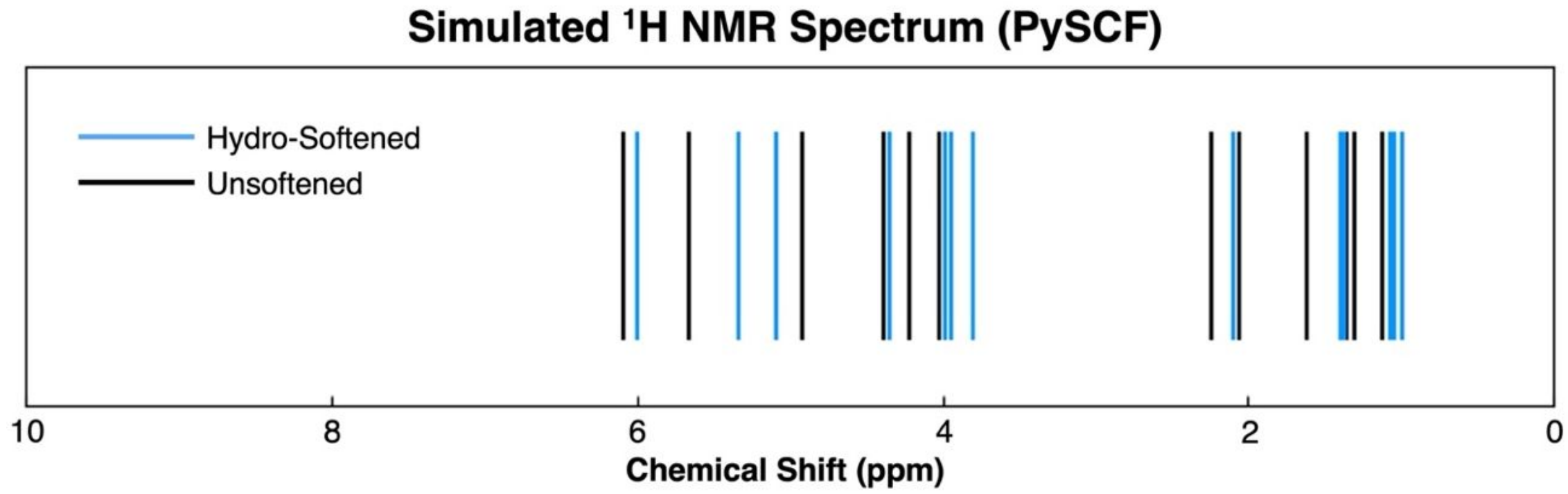

Figure S4. Simulated ^{1}H NMR spectra for unsoftened (black) and hydro-softened chitosan (blue). Stick spectra show computed chemical shifts (ppm) for hydrogen atoms in unsoftened chitosan and hydro-softened chitosan with one water molecule, obtained via density functional theory (DFT) and gauge-including atomic orbital (GIAO) calculations using PySCF.

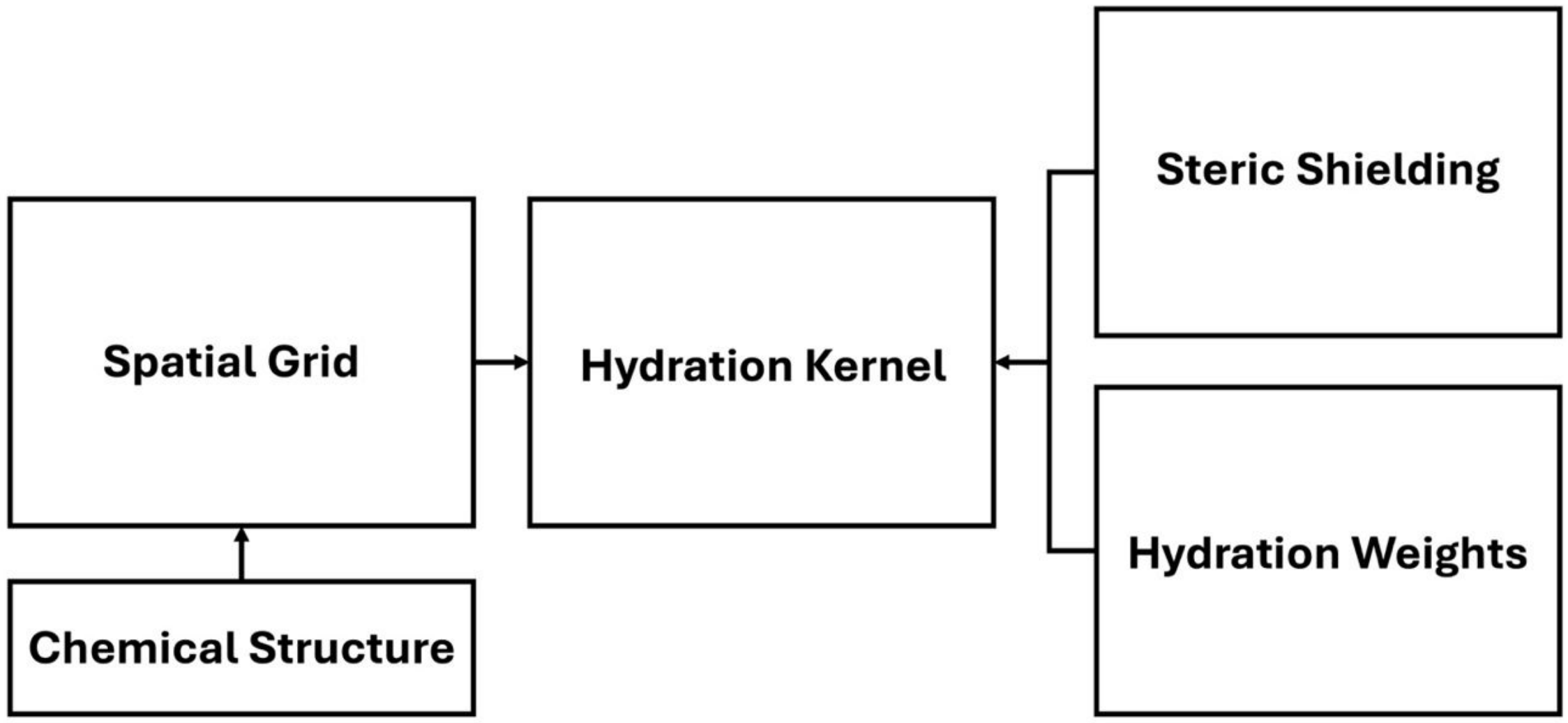

Figure S5. Schematic workflow of the voxelized hydration field simulation informed by steric shielding and functional group weighting.

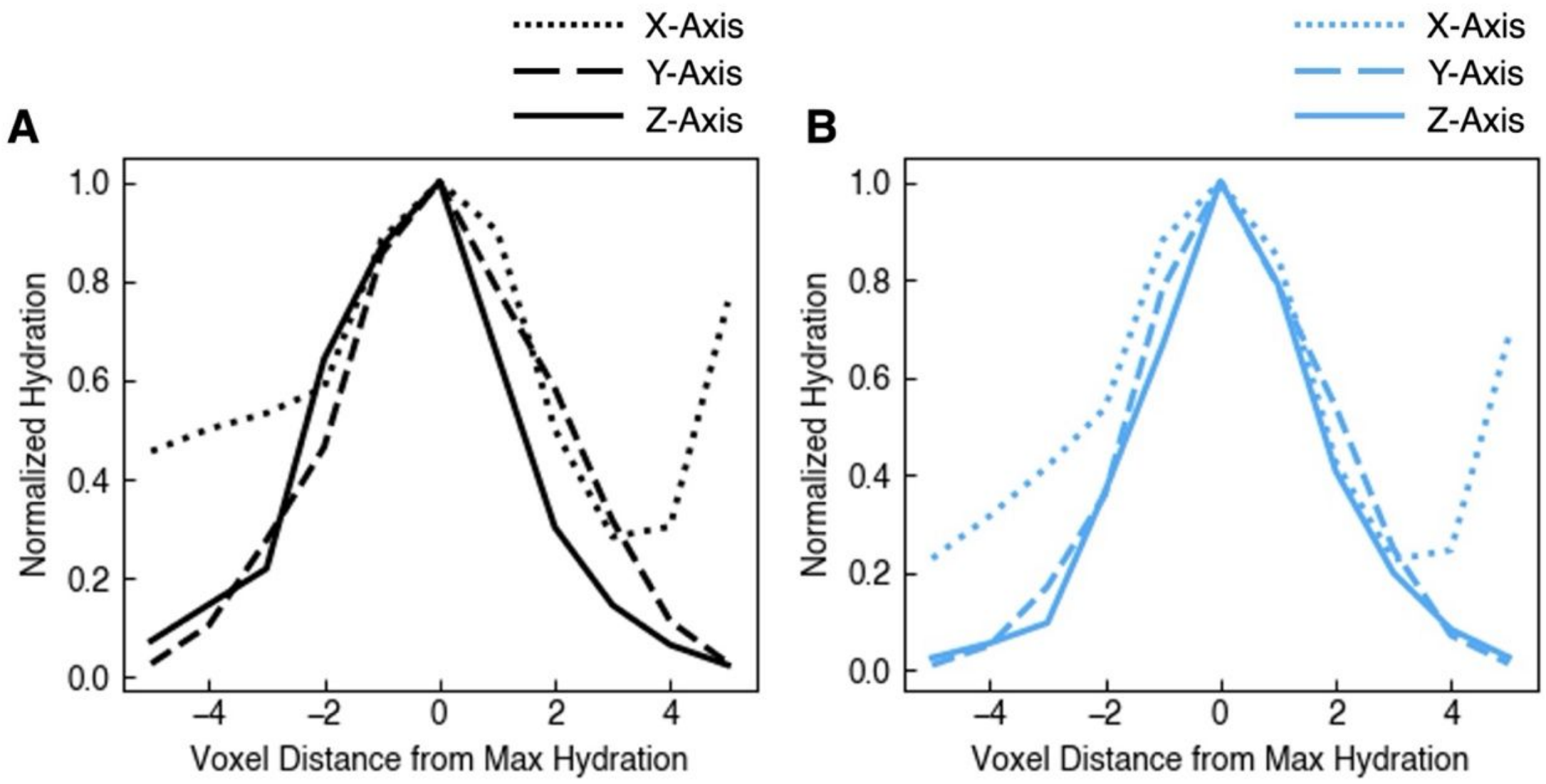

Figure S6. Hydration anisotropy profiles. Normalized hydration intensities along X, Y, and Z axes, plotted as a function of voxel distance from the site of maximal hydration. Compared to the unsoftened sample **(A)**, the hydro-softened **(B)** shows steeper and more symmetric decay across all axes, indicating enhanced structural confinement of water. This anisotropic distribution supports the emergence of directionally restricted, low-entropy hydration domains as a hallmark of hydro-softening.

A

	Chitosan	Water	HCl	TEOS
20%	1 g	100 mL	6.2 mL	260 μL
40%				520 μL
60%				780 μL
80%				1040 μL

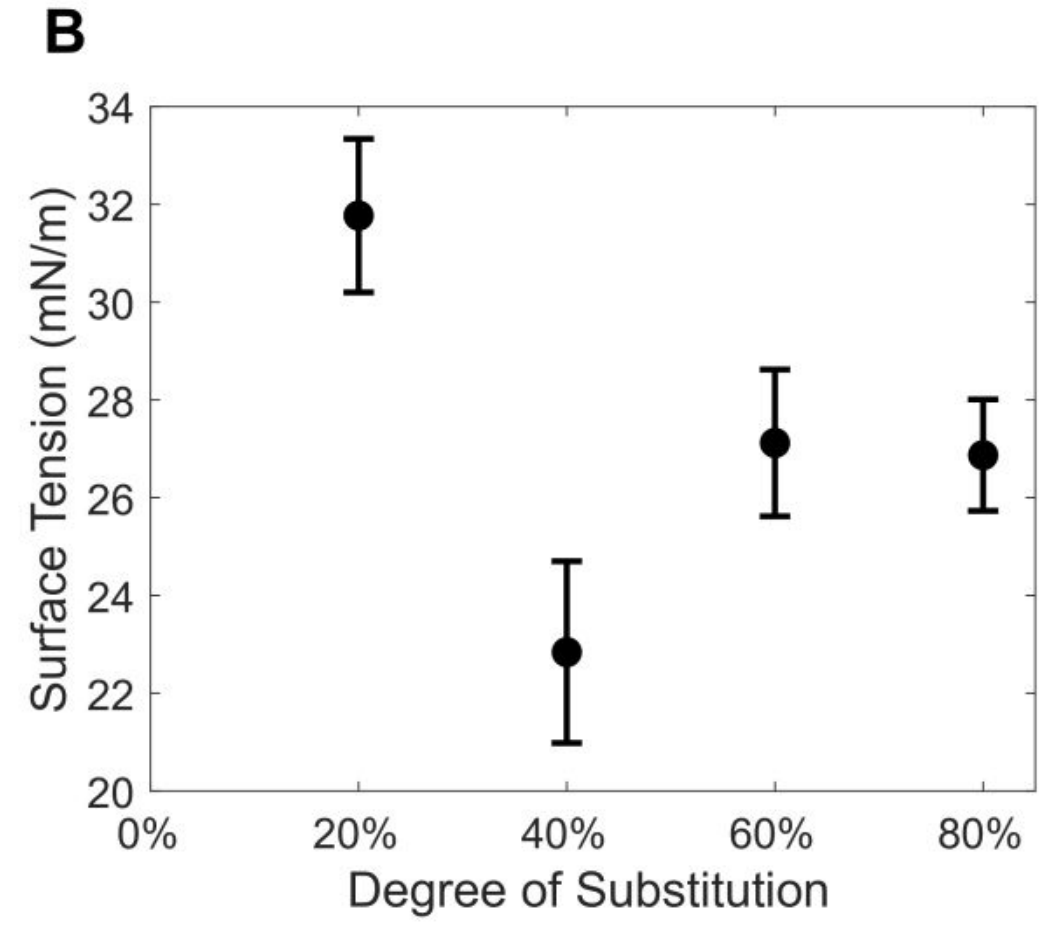

Figure S7. Surface tension and degree of silanization. **(A)** Formulation ratios for a parameter sweep of chitosan-TEOS degree of substitution. **(B)** With ~22 mN/m, chitosan balances low surface tension for improved spreading on high-energy substrates like PDMS at 40% degree of substitution, while maintaining hydroxyl groups for effective condensation and water expulsion.

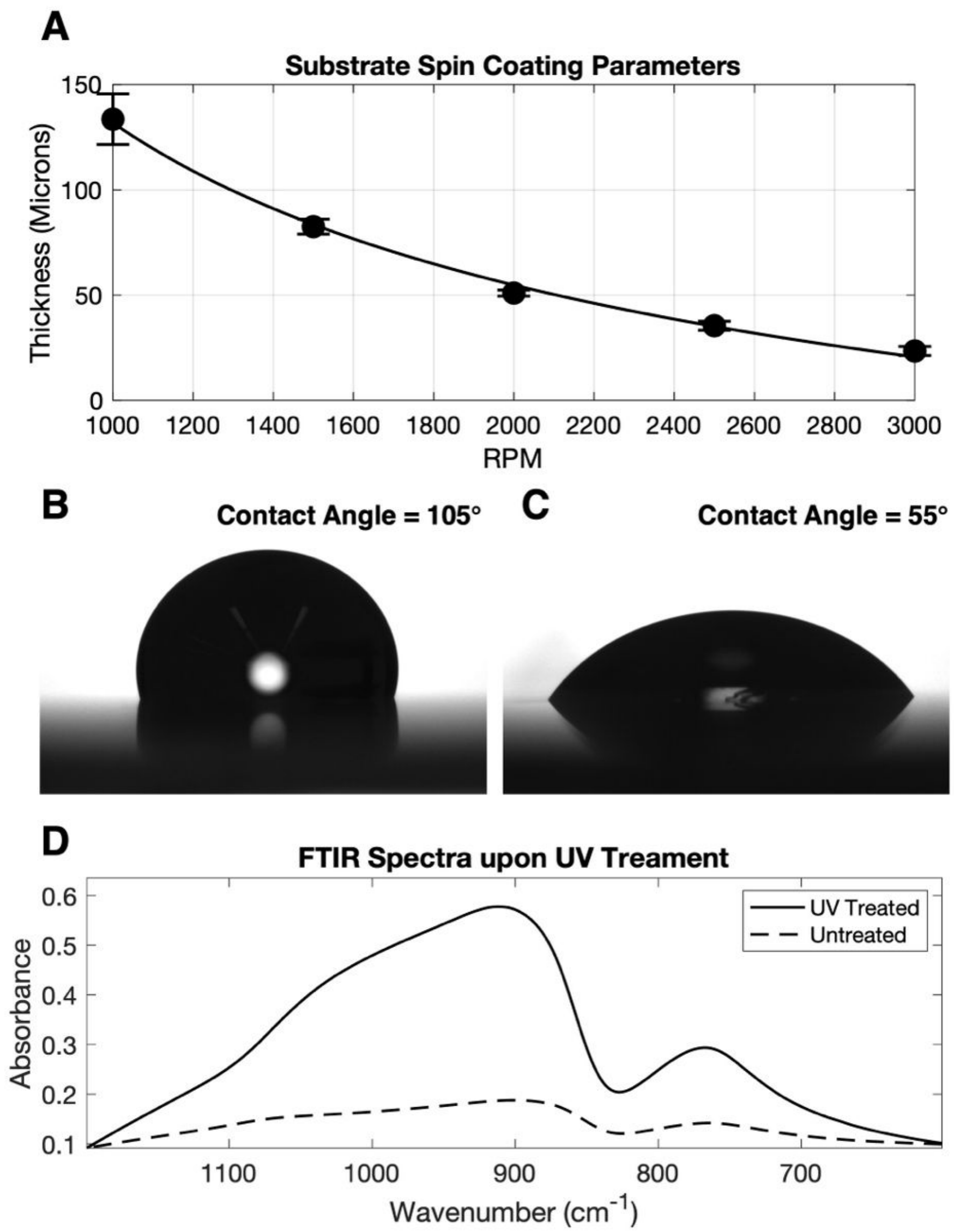

Figure S8. Spin coating and UV-O_3 treatment considerations. **(A)** Dry thickness measurement of the spin-coated PDMS substrates against the spin-rates. **(B)** Water contact angle measurement before UV-O_3 treatment, revealing pronounced hydrophobicity. **(C)** Water contact angle measurement post-UV-O_3 treatment. **(D)** FTIR spectra confirming the introduction of hydroxyl groups to the PDMS, resulting in reduced surface energy upon coating.

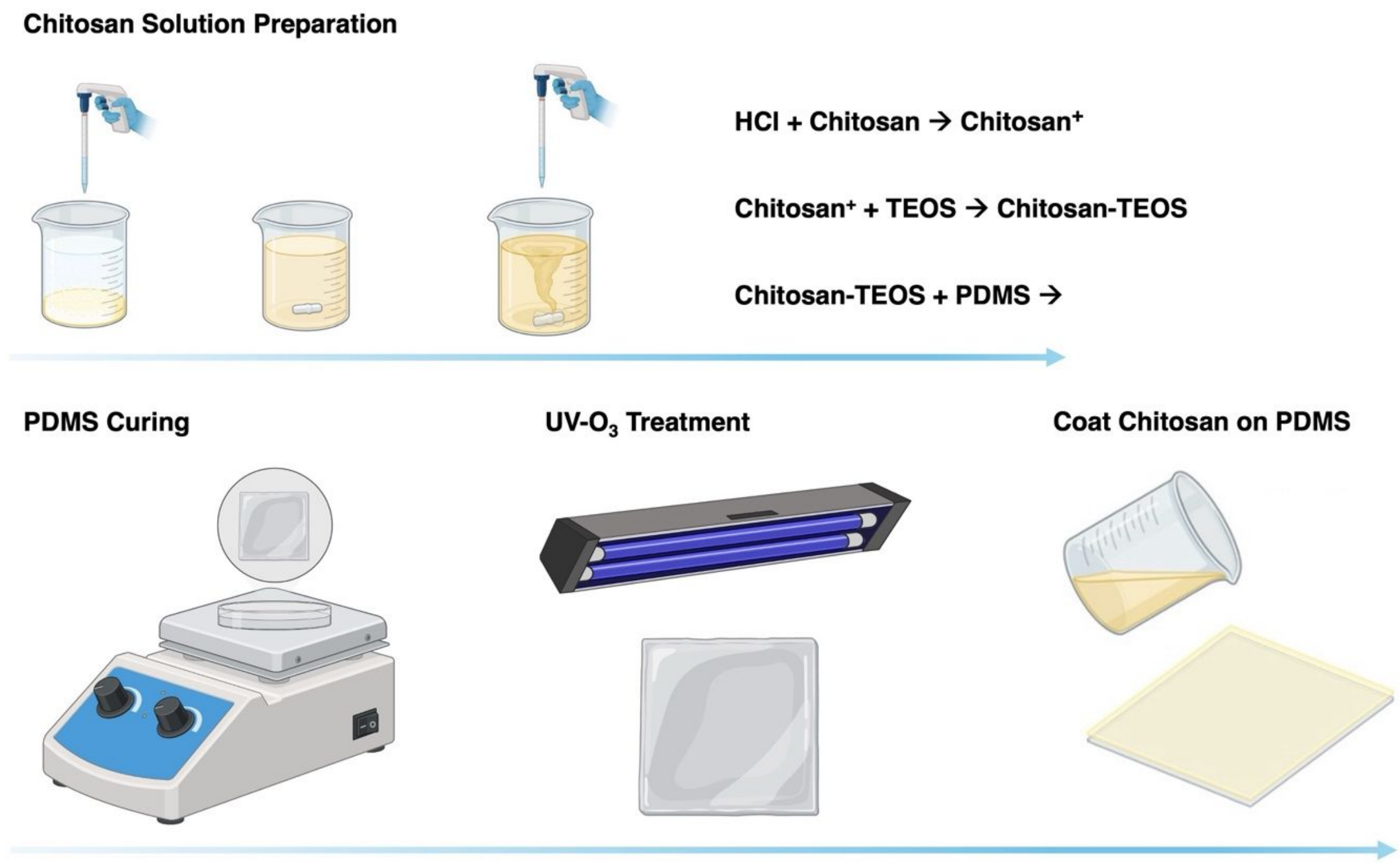

Figure S9. Fabrication schematic. Chitosan is dissolved and protonated using dilute HCl, then hydrolyzed with a silane precursor. The siloxane is treated with UV-O_3 to introduce hydroxyl groups. The resulting condensation facilitates the adsorption of the chitosan onto the substrate.

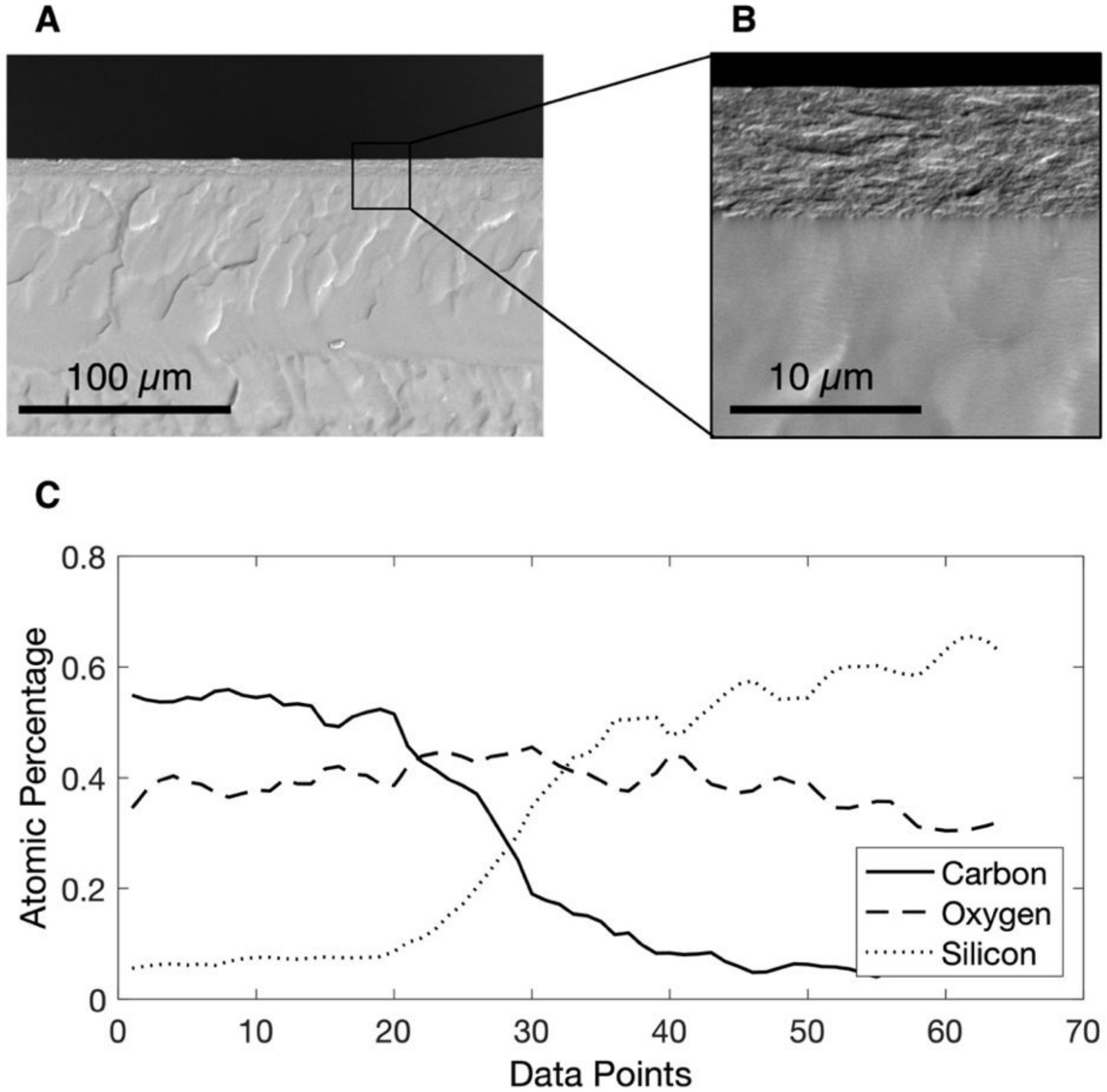

Figure S10. Cross-sectional profile of the substrate-chitosan interface. **(A)** Freeze-fractured cross-section SEM image shows that a thin (10 µm) chitosan film sits on a thick substrate (200 µm). **(B)** Focused SEM cross-section profile illustrating a uniform coating on the surface. **(C)** Energy dispersive spectroscopy (EDS) indicates shifting atomic percentages across the cross-section.

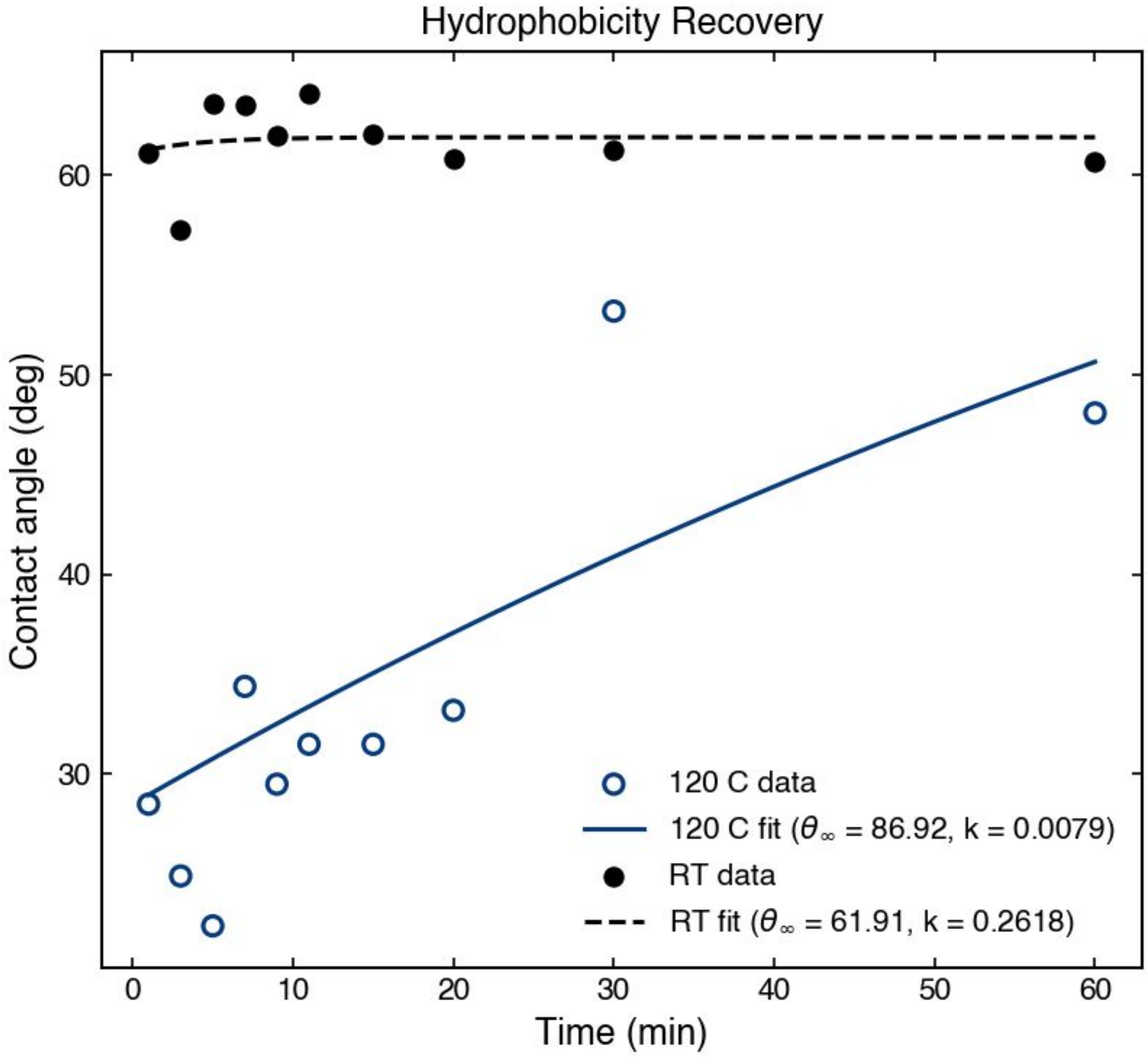

Figure S11. Hydrophobic recovery of PDMS substrates under processing temperatures.

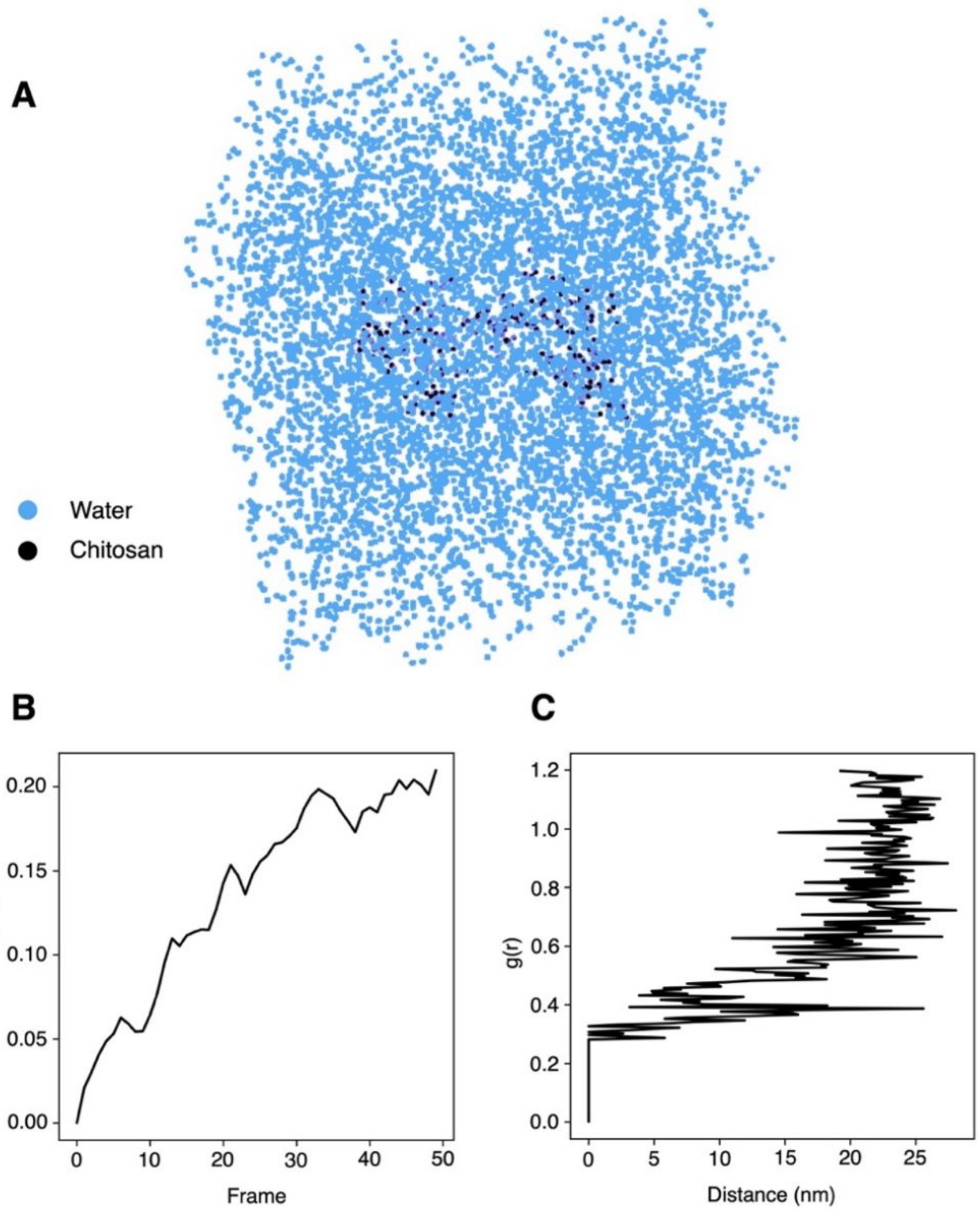

Figure S12. MD simulation. **(A)** MD simulation snapshot of chitosan (black) in a periodic box of water molecules (hydration blue). **(B)** Root-mean-square deviation (RMSD) of chitosan over time, indicating the dynamic flexibility in response to hydration. **(C)** Radial distribution function g(r) of water around chitosan. g(r) denotes the normalized local density of water at a distance r from the polymer relative to the bulk water density. The gradual increase reflects broad, diffuse hydration rather than discrete solvation shells.

Table S1. Heuristically assigned hydration weights in the hydration kernel model.

Atom Type	Hydration Weights
Oxygen (O)	1.0
Nitrogen (N)	1.4
Carbon (C)	0.3
Hydrogen (H)	0.1

Table S2. Chitosan (CS) Purity and reported elastic moduli values.

Process	Materials	CS Atomic Weight %	Elastic Modulus	References
Blending	CS-PTTG	80	300 MPa	Khankhuean et al.[10]
Blending	CS-Honey	80	500 MPa	Madian et al.[11]
Plasticization	CS-DES	40	60 MPa	Jakubowska et al.[12]
Plasticization	CS-GLY	80	140 MPa	Sabbah et al.[13]
Hydrogel	CS-PBS	12	120 kPa	da Silva et al.[14]
Hydrogel	CS-NaOH	12	240 kPa	Ben Bouali et al.[15]
Copolymerization	CS/PTMC-co-TMC-COOH	75	133 MPa	Khankhuean et al.[10]
Copolymerization	CS/PTMC-co-TMC-COOH/mGlu	75	126 MPa	Khankhuean et al.[10]
Composite	PCL/CS/PCL Sandwich	30	2.2 GPa	Aryaei et al.[16]
Pure Chitosan	CS	100	3.3 GPa	Aryaei et al.[16]

Table S3. Substrate and film thickness at various substrate curing conditions.

Thickness(mm)/PDMS Curing Condition (°C)	RT	40	80	120
PDMS	0.963 ± 0.06	0.975 ± 0.08	1.01 ± 0.10	1.03 ± 0.04
CS/PDMS	1.03 ± 0.06	1.06 ± 0.05	1.065 ± 0.06	1.07 ± 0.06
CS Layer	0.069 ± 0.06	0.083 ± 0.07	0.053 ± 0.08	0.046 ± 0.05

Table S4. Time-resolved contact angle measurements of PDMS substrates following UV-O_3 treatment.

Contact Angle Measurement After Two-hour UV-O_3 Treatment (°)				
PDMS Substrate Processed at 120°C (Condition for hydro-softening samples)				
Time Elapsed	Reading 1 (Left)	Reading 2 (Center)	Reading 3 (Right)	Average
1 min	30.55	26.48	28.51	28.51
3 min	24.5	25.38	24.94	24.94
5 min	23.8	20.98	22.39	22.39
7 min	35.15	33.7	34.42	34.42
9 min	27.23	31.7	29.46	29.46
11 min	31.08	31.99	31.54	31.54
15 min	33.95	29.12	31.53	31.53
20 min	30.76	35.73	33.24	33.24
30 min	52.68	53.86	53.12	53.22
60 min	47.5	48.64	48.07	48.07
PDMS Substrate Processed at RT (Condition for unsoftened samples)				
Time Elapsed	Reading 1 (Left)	Reading 2 (Center)	Reading 3 (Right)	Average
1 min	61.82	60.47	61.14	61.14
3 min	58.43	56.21	57.32	57.32
5 min	64.2	62.52	64.1	63.61
7 min	64.13	62.93	63.53	63.53
9 min	66.67	57.39	62.03	62.03
11 min	63.52	64.61	64.06	64.06

**15 min**	60.14	67.48	58.81	62.14
**20 min**	60.76	60.91	60.84	60.83
**30 min**	63.71	59.56	61.36	61.54
**60 min**	60.9	60.41	60.66	60.66

Table S5. Contact angle measurements of untreated PDMS substrates.

Untreated PDMS Substrates (°)				
Sample	Reading 1	Reading 2	Reading 3	Average
Substrate Processed at 120C	93.57	94.25	93.91	93.91
Substrate Processed at RT	104.88	105.08	102.30	104.08

Supporting Information References

(1) Vinogradova, O. I.; Feuillebois, F. Interaction of Elastic Bodies via Surface Forces. 1. Power-Law Attraction. *Langmuir* **2002**, *18* (13), 5126-5132. DOI: 10.1021/la011726r.

(2) Churaev, N. V. Derjaguin's disjoining pressure in the colloid science and surface phenomena. *Advances in Colloid and Interface Science* **2003**, *104* (1), xv-xx. DOI: https://doi.org/10.1016/S0001-8686(03)00032-0.

(3) Landau, L. D. L., E. M. *Theory of Elasticity*; Elsevier Butterworth-Heinmann, 1986.

(4) Mondal, J.; Morrone, J. A.; Berne, B. J. How hydrophobic drying forces impact the kinetics of molecular recognition. *Proceedings of the National Academy of Sciences* **2013**, *110* (33), 13277-13282. DOI: 10.1073/pnas.1312529110 (acccessed 2026/02/23).

(5) Eastman, P.; Swails, J.; Chodera, J. D.; McGibbon, R. T.; Zhao, Y.; Beauchamp, K. A.; Wang, L. P.; Simmonett, A. C.; Harrigan, M. P.; Stern, C. D.; et al. OpenMM 7: Rapid development of high performance algorithms for molecular dynamics. *PLoS Comput Biol* **2017**, *13* (7), e1005659. DOI: 10.1371/journal.pcbi.1005659 From NLM.

(6) Jorgensen, W. L.; Chandrasekhar, J.; Madura, J. D.; Impey, R. W.; Klein, M. L. Comparison of simple potential functions for simulating liquid water. *The Journal of Chemical Physics* **1983**, *79* (2), 926-935. DOI: 10.1063/1.445869 (acccessed 7/6/2025).

(7) Boothroyd, S.; Behara, P. K.; Madin, O. C.; Hahn, D. F.; Jang, H.; Gapsys, V.; Wagner, J. R.; Horton, J. T.; Dotson, D. L.; Thompson, M. W.; et al. Development and Benchmarking of Open Force Field 2.0.0: The Sage Small Molecule Force Field. *Journal of Chemical Theory and Computation* **2023**, *19* (11), 3251-3275. DOI: 10.1021/acs.jctc.3c00039.

(8) Mobley, D. L.; Bannan, C. C.; Rizzi, A.; Bayly, C. I.; Chodera, J. D.; Lim, V. T.; Lim, N. M.; Beauchamp, K. A.; Slochower, D. R.; Shirts, M. R.; et al. Escaping Atom Types in Force Fields Using Direct Chemical Perception. *J Chem Theory Comput* **2018**, *14* (11), 6076-6092. DOI: 10.1021/acs.jctc.8b00640 From NLM.

(9) Humphrey, W.; Dalke, A.; Schulten, K. VMD: Visual molecular dynamics. *Journal of Molecular Graphics* **1996**, *14* (1), 33-38. DOI: https://doi.org/10.1016/0263-7855(96)00018-5.

(10) Khankhuean, A.; Morimura, Y.; Ajiro, H. Improving the mechanical properties of chitosan through blending with poly(trimethylene carbonate) copolymer. *International Journal of Biological Macromolecules* **2024**, *283*, 137830. DOI: https://doi.org/10.1016/j.ijbiomac.2024.137830.

(11) Madian, N. G.; El-Ashmanty, B. A.; Abdel-Rahim, H. K. Improvement of Chitosan Films Properties by Blending with Cellulose, Honey and Curcumin. *Polymers* **2023**, *15* (12), 2587.
(12) Jakubowska, E.; Gierszewska, M.; Nowaczyk, J.; Olewnik-Kruszkowska, E. Physicochemical and storage properties of chitosan-based films plasticized with deep eutectic solvent. *Food Hydrocolloids* **2020**, *108*, 106007. DOI: https://doi.org/10.1016/j.foodhyd.2020.106007.
(13) Sabbah, M.; Di Pierro, P.; Cammarota, M.; Dell'Olmo, E.; Arciello, A.; Porta, R. Development and properties of new chitosan-based films plasticized with spermidine and/or glycerol. *Food Hydrocolloids* **2019**, *87*, 245-252. DOI: https://doi.org/10.1016/j.foodhyd.2018.08.008.
(14) da Silva, R. L. C. G.; Ando, R. A.; Petri, D. F. S. Unveiling the Effect of Aqueous-Phase Dynamics on Chitosan Hydrogel Film Mechanical Properties Through AFM Nanoindentation and Tensile Testing. *Gels* **2025**, *11* (7), 496.
(15) Ben Bouali, A.; Montembault, A.; David, L.; Von Boxberg, Y.; Viallon, M.; Hamdi, B.; Nothias, F.; Fodil, R.; Féréol, S. Nanoscale mechanical properties of chitosan hydrogels as revealed by AFM. *Prog Biomater* **2020**, *9* (4), 187-201. DOI: 10.1007/s40204-020-00141-4 From NLM.
(16) Aryaei, A.; Jayatissa, A. H.; Jayasuriya, A. C. Nano and micro mechanical properties of uncross-linked and cross-linked chitosan films. *J Mech Behav Biomed Mater* **2012**, *5* (1), 82-89. DOI: 10.1016/j.jmbbm.2011.08.006 From NLM.
